# Exploring the diversity and genomics of cultivable *Bacillus*-related endophytic bacteria from the medicinal plant *Galium aparine* L.

**DOI:** 10.3389/fmicb.2025.1612860

**Published:** 2025-06-30

**Authors:** Natalia Rutkowska, Maurycy Daroch, Olga Marchut-Mikołajczyk

**Affiliations:** ^1^Institute of Molecular and Industrial Biotechnology, Lodz University of Technology, Łódź, Poland; ^2^School of Environment and Energy, Peking University Shenzhen Graduate School, Shenzhen, China

**Keywords:** bacterial endophytes, *Galium aparine* L., phylogenetics, genome mining, mobile genetic elements, secondary metabolites, plant microbiome

## Abstract

**Introduction:**

Endophytes are crucial partners that contribute to the plants’ health and overall wellbeing. Apart from the elucidation of the relationship between plants and their microbiota, the metabolic potential of endophytes is also of a special interest. Therefore, it is crucial to isolate and taxonomically identify endophytes, as well as to investigate their genomic potential to determine their significance in plant health and potential as bioactive metabolite producers for industrial application.

**Methods:**

In this study, we isolated ten endophytic bacterial strains from different tissues of medicinal plant *Galium aparine* L. and performed *de novo* assembly of their genomes using short and long reads. Comparative genomic analysis was conducted to assess the accurate taxonomic identification of the strains. The investigation also focused on the presence of mobile genetic elements and their significance concerning endophytic lifestyles. We performed functional annotation of coding sequences, particularly targeted genes that encode carbohydrate enzymes and secondary metabolites within gene clusters.

**Results:**

Through sequencing using two complementary methods, we obtained 10 bacterial genomes, ranging in size, coding density and number of mobile genetic elements. Our findings provide a first insight into the cultivable bacterial community of the medicinal plant *Galium aparine* L., their genome biology, and potential for producing valuable bioactive metabolites. Obtained whole genome sequences allowed for complete phylogenetic analysis, which revealed crucial insights into the taxonomic status of bacteria and resulted in the discovery of two putatively novel bacterial species from the *Bacillus* and *Priestia* genera, suggesting that plants are hiding a reservoir of novel species with potentially useful properties and unknown mechanisms related to their relationship with plant host.

## Introduction

Plants have developed complex and diverse relationships with microorganisms in their neighborhood as they co-exist in the same ecological niche, constantly subjected to selection pressures. Endophytic microorganisms are an exceptional group of plant-associated microorganisms, as they thrive inside their tissues without causing any detrimental effect or disease to the host (Hardoim et al., 2015). The relationship between plants and their endophytes is mutually beneficial. The plant provides a safe niche less disturbed by fluctuating conditions (e.g., temperature amplitude) and a constant supply of nutrients, while the endophytes provide positive effects on plant health and fitness, therefore ensuring their own survival (Afzal et al., 2019). Among the beneficial traits exhibited by endophytes, the most common ones are nitrogen fixation and phosphorus solubilization, which enhance the uptake of these crucial nutrients by a plant. Additionally, endophytes produce various bioactive substances such as plant hormones (indole-3-acetic acid, gibberellins, abscisic acid, etc.) siderophores, antibiotics, and insecticides (Afzal et al., 2019; Egamberdieva et al., 2017; Eid et al., 2021). Medicinal plants are particularly intriguing for the identification of their endophytes, especially bacteria, due to the abundant production of various chemicals with therapeutic qualities. However, the investigation of these bacterial endophytes has been limited (Sharma et al., 2023). Existing evidence proves that endophytes are capable of producing *ex planta* bioactive compounds that are identical or very similar to those of their host plants; therefore, this concept may 1 day be applied as technology of their large-scale production (Kusari and Spiteller, 2011; Rutkowska et al., 2023).

A broad group of endophytes, classified as “facultative,” enters the plant via root or stem cracks, previously being rhizospheric or epiphytic microorganisms, respectively. Another type, known as “obligate,” maintains an intrinsic bond with its host throughout its entire life cycle and typically transmits itself vertically through seeds (Rosenblueth and Martínez-Romero, 2006). Seed microbiota is transmitted by plants across generations, being the starting point for community assembly in the new seedling (Semenzato et al., 2022a; Truyens et al., 2015). Obligate endophytes are recalcitrant or even impossible to grow outside plants in laboratory conditions; thus, cultivable endophytes are considered to be mostly facultative (Hardoim et al., 2008; Papik et al., 2020). Members of the *Bacillaceae* family, especially *Bacillus* species, are among the most commonly encountered cultivable endophytic bacteria. Their plant growth-promoting activities and versatility for industrial use have led to extensive research in recent decades (Jasim et al., 2016; Lopes et al., 2018; Marchut-Mikolajczyk et al., 2018, 2020; Marchut-Mikołajczyk et al., 2023; Zhou et al., 2021).

Apart from *ex planta* studies of isolated endophytes, modern omics approaches conjoining different aspects of single endophyte interaction with the host plant and other inhabitants are crucial to deciphering not only their role in host plant ecology, but also the impact of it on endophytes’ functioning. As the plant itself represents a peculiar niche that requires specific adaptations from its inhabitants, endophytes influence the plant environment and contribute to its secondary metabolism as well. Kaul et al. (2016) broadly discussed methodologies essential for resolving some of the issues, including genomics, transcriptomics, and proteomics. Comparative genomics provides insights into mechanisms steering the endophytic lifestyle, such as colonization patterns, plant growth promotion, induced resistance, interactions with other associated microorganisms, as well as the secondary metabolism of an endophyte (Kaul et al., 2016; Pinski et al., 2019). The conventional approach to screening for novel compounds relies on biological assays based on their putative activity; however, this approach is time- and cost-consuming and often results in rediscovery of known compounds (Kenshole et al., 2021). Therefore, in the last years, there has been a shift toward a genome mining approach, which takes advantage of recent advancements in DNA sequencing technologies and bioinformatics. This reveals the hidden potential of cultivable endophytes to produce specific compounds (enzymes, non-ribosomal peptides, polyketides), which are often not expressed under standard laboratory conditions. This knowledge could guide future cultivation methods and approaches to activate genes responsible for their synthesis (Kenshole et al., 2021). All of those endophytic bioactive compounds can be successfully used in pharmaceutical and other industrial applications, as detergents (Marchut-Mikolajczyk et al., 2018), biofuels (Zhang et al., 2014), biopesticides (Rong et al., 2020) or pigments (Hagaggi and Abdul-Raouf, 2023), as well as in agriculture and crop production (Eid et al., 2021; Lopes et al., 2018).

To our knowledge, this is the first report on endophytic bacteria isolated from *Galium aparine* L., a medicinal herb widely growing in Europe, North America and Asia; known for its beneficial effects on kidneys, skin disorders, wounds, high blood pressure, and insomnia (Al-Snafi, 2018; Ilina et al., 2019). While there is no prior published record on endophytes, both bacterial and fungal, a research by Bemmann and Kulle (1988) reported the presence of rhizospheric fungi belonging to the genera *Aspergillus*, *Mucor*, *Penicillium*, and *Rhizophus* (Bemmann and Kulle, 1988). All plants developed close associations with surrounding microorganisms, as their support is crucial for plants as sessile organisms to adapt effectively to changing environmental conditions. However, many plant have yet to be studied in terms of their microbiome (Rutkowska et al., 2023). The study aimed to (1) isolate cultivable endophytic bacteria from the tissues of *Galium aparine* L., (2) obtain their genomic sequences and identify them taxonomically, (3) study the presence of mobile genetic elements as drivers of evolution and (4) mine their genomes for encoded bioactive compounds with beneficial uses in agriculture and other industries.

## Results and discussion

### Isolation of endophytic bacteria

From the surface-sterilized fragments of leaves, stems and roots of *Galium aparine* L., a total of 10 bacterial isolates were isolated and purified by subsequent streak plating. The effectiveness of the surface sterilization method was confirmed by the lack of microbial growth on the control plates containing water from the plant’s last rinsing after 7 days of incubation. Obtained strains were named as G followed by R, S, or L for roots, stem and leaves, respectively.

### Genome sequencing and assembly

Whole genome sequencing (WGS) was performed, followed by *de novo* hybrid assembly and annotation (Table 1). The assembly size of 10 isolated strains ranges from 4.17 (GS2) to 6.10 Mbp (GL1). The average genome-wide GC content varies between 34.9% and 40.5%. The number of assembled contigs varied from sample to sample, but of all the datasets, only the GR2 genome is assembled to the complete chromosome level. The strains differ in the number of genes. The obtained results ensure good quality assembly without contamination.

**TABLE 1:** Genome assembly statistics and annotation features of endophytic bacteria from *Galium aparine* L.

Feature	Strain name									
	GR1	GR2	GR3	GR4	GS1	GS2	GS3	GL1	GL2	GL3
Size (Mbp)	5.66	5.63	5.97	5.69	5.94	4.17	5.70	6.10	5.70	5.99
GC content (%)	35.23	40.48	34.87	37.70	34.86	37.69	35.05	35.03	35.12	35.11
Contigs	18	1	10	45	58	9	26	19	21	8
N50 (Mbp)	4.99	5.63	3.13	0.91	0.61	3.77	2.92	2.13	3.78	5.29
L50	1	1	1	3	4	1	1	2	1	1
Genes (all)	5,840	5,392	6,085	5,937	6,061	4,285	5,803	6,307	5,808	6,132
CDSs*	5,716	5,261	5,941	5,842	5,986	4,157	5,676	6,173	5,659	5,982
rRNAs*	19	42	34	11	10	27	28	27	38	39
tRNAs*	100	83	105	76	60	96	94	102	106	106
ncRNAs*	5	6	5	8	5	5	5	5	5	5
Completeness (%)	99.41	99.35	99.11	99.27	99.27	99.35	99.22	98.24	99.22	98.82
Contamination (%)	0.00	0.32	0.06	0.65	0.97	0.00	0.43	0.10	0.43	0.25
Complete BUSCOs (%)	99.8	99.8	99.5	100.0	99.8	99.6	99.8	99.3	99.8	99.8

*CDS, coding sequence; ncRNA, non-coding RNA; rRNA, ribosomal RNA, tRNA, transfer RNA.

The genome sequences are deposited in the National Center for Biotechnology Information (NCBI, United States) database under the BioProject number PRJNA1068863.

### Taxonomic identification

The initial attempt at identification was conducted by manually extracting 16S rDNA and *gyrA* sequences from annotated genomes, followed by their comparison against the NCBI database using BLASTN (data not shown). However, for most of the strains, this approach was highly inconclusive as the percentage of identity in gene sequences was the same for several species, a common occurrence within the *Bacillus* genus (Liu et al., 2022). What is more, many members of *Bacillus cereus* group members are identified solely based on their 16S rDNA sequences, leading to further misidentifications in the NCBI database. As a consequence, taxonomic identification of isolated strains was based on whole genome sequence analysis using Type (Strain) Genome Server (TYGS), which calculates digital DNA:DNA hybridization (dDDH) parameter values for *in silico* species delineation, considering only verified type strains from the List of Prokaryotic names with Standing in Nomenclature (LPSN) (Meier-Kolthoff et al., 2022; Tindall et al., 2010). So-called “reference” or “representative” strains are not reliable and their use in identification may lead to further taxonomic misidentification (Meier-Kolthoff et al., 2022). Also, the Average Nucleotide Identity (ANI) values, the second parameter for species delineation, were calculated. The proposed identification of the isolates with ANI and dDDH is presented in Table 2.

**TABLE 2:** Whole genome sequencing (WGS)-based identification of isolated strains.

Strain	Proposed identification (NCBI GenBank Assembly Accession no.)	Closest type-strain match (NCBI GenBank Assembly Accession no.)	ANI (%)*	dDDH (%)*
GR1	*Bacillus pretiosus* GR1 (GCA_036408975.1)	*Bacillus pretiosus* SAIUCEU11T(GCA_025916425.1)	97.14	78.20
GR2	*Peribacillus frigoritolerans* GR2 (GCA_036352075.1)	*Peribacillus frigoritolerans* DSM 8801 (GCA_024169475.1)	97.24	80.30
GR3	*Bacillus cereus* GR3 (GCA_036408805.1)	*Bacillus cereus* ATCC 14579 (GCA_006094295.1)	97.16	74.10
GR4	*Priestia megaterium* GR4 (GCA_036350305.1)	*Priestia megaterium* ATCC 14581 (GCA_017086525.1)	96.91	72.80
GS1	*Bacillus thuringiensis* GS1 (GCA_036350635.1)	*Bacillus thuringiensis* ATCC 10792 (GCA_000161615.1)	96.52	69.10
GS2	*Priestia* sp. GS2 (GCA_036409025.1)	*Priestia flexa* NBRC 15715 (GCA_001591565.1)	92.40	47.40
GS3	*Bacillus cereus* GS3 (GCA_036408815.1)	*Bacillus cereus* ATCC 14579 (GCA_006094295.1)	98.30	83.40
GL1	*Bacillus* sp. GL1 (GCA_039680825.1)	*Bacillus wiedmannii* FSL W8-0169 (GCA_001583695.1)	95.06	59.80
GL2	*Bacillus cereus* GL2 (GCA_036408835.1)	*Bacillus cereus* ATCC 14579 (GCA_006094295.1)	98.30	83.50
GL3	*Bacillus wiedmannii* GL3 (GCA_036350675.1)	*Bacillus wiedmannii* FSL W8-0169 (GCA_001583695.1)	96.63	70.00

*ANI, Average Nucleotide Identity; dDDH, digital DNA-DNA hybridization.

**FIGURE 1:**
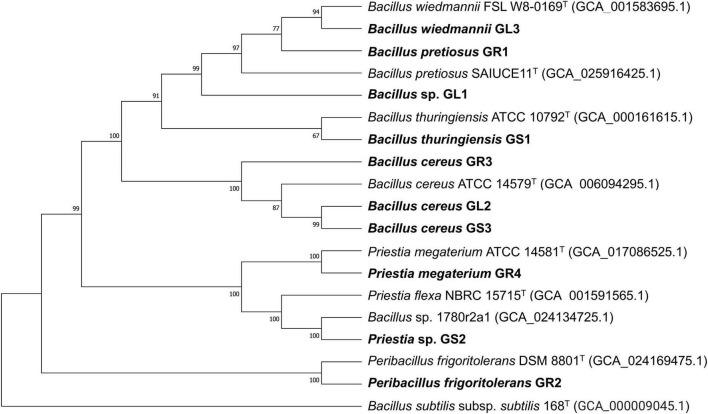
Phylogenetic tree of *Galium aparine* L’s endophytes and their closest type-strain matches based on whole genome alignments. Single nucleotide polymorphisms (SNPs) and indels within the multiple sequence alignments were constructed by the reference sequence Alignment based Phylogeny builder (REALPHY) and extracted for subsequent phylogeny reconstruction using MEGA v11.0.9 by the Neighbor-joining method, with a bootstrap of 1,000 replications (Bertels et al., 2014; Tamura et al., 2021). Bootstrap confidence levels are indicated at the internodes.

The majority of the isolates surpassed thresholds recognized as the cut-offs for species delineation, ≥ 95%–96% for ANI and ≥ 70% for dDDH (Auch et al., 2010; Richter and Rosselló-Móra, 2009). As stated in Table 2, most of the isolated species belong to the *Bacillus* genus or are closely related. Strains can be divided into three groups (Figure 1): (1) *Priestia* spp. – *Priestia* sp. GS2, *Priestia megaterium* GR4, (2) *Peribacillus frigoritolerans* GR2 and (3) *Bacillus cereus* group – *Bacillus thuringiensis* GS1, *Bacillus cereus* GS3, GL2, and GR3, *Bacillus wiedmannii* GL3, *Bacillus pretiosus* GR1, and *Bacillus* sp. GL1.

Among the strains isolated from *Galium aparine* L., two strains, GS2 and GL1, showed the possibility of being novel strains, as ANI and dDDH values are too low to classify them into known species (Table 2). Their taxonomic identification was determined to be *Priestia* sp. and *Bacillus* sp., respectively. Surprisingly, based on manual comparisons of the two strains, *Priestia* sp. GS2 displayed high similarity to *Bacillus* sp. 1708r2a1 (NCBI GenBank assembly no. GCA_024134725.1), which was isolated from the clean room of NASA Center with ANI and dDDH values reaching 99.48% and 95.70%, respectively. In contrast, these parameters are below threshold established for the closest type strain *Priestia flexa* NBRC 15715 (NCBI GenBank assembly no. GCA_001591565.1).

Firmicutes, with *Bacillus* as the most representative genus, is one of the most prevalent phyla among culturable endophytic bacteria. *Bacillus* spp. endophytes are known for their plant growth potential and often demonstrate antibacterial and antifungal activities (Lopes et al., 2018). They are very abundant in soil; thus, they probably colonize the plant via roots attracted by their exudates. *Bacillus* genus was known for its heterogeneity, which was a result of loose classification criteria in the past, by which the capability of forming spores in the presence of oxygen was sufficient (Logan et al., 2009). A total of 2020 was a significant year for *Bacillus* genus taxonomy (Gupta et al., 2020; Patel and Gupta, 2020). First, six new genera of *Bacillus* species were proposed after a broad analysis of 1,172 core *Bacillaceae* proteins and the identification of conserved signature indels (CSIs) for each – *Peribacillus*, *Cytobacillus*, *Mesobacillus*, *Neobacillus*, *Metabacillus*, and *Alkalihalobacillus* (Patel and Gupta, 2020). To *Peribacillus* spp., species such as *Bacillus muralis*, *butanolivorans*, and *simplex*, have been transferred due to the three CSIs (HAMP domain-containing protein, phosphor-N-acetylmuramoyl pentapeptide-transferase and stage II sporulation protein E). *Peribacillus frigotolerans* has come an even longer way to proper identification, as it was first classified as *Brevibacterium frigoritolerans*, then reclassified as *Bacillus frigoritolerans* (Liu et al., 2020; Montecillo and Bae, 2022a). Later, another 17 distinct *Bacillus* species clades were distinguished based on CSIs, which included the separation of *Priestia* genus (*megaterium* clade), to which *Bacillus abyssalis, aryabhattai, endophyticus, filamentosus, flexus, koreensis*, and *megaterium* were transferred as they all contain two CSIs in the protein oligoribonuclease NrnB (DHH superfamily) (Gupta et al., 2020).

Although the *Bacillus subtilis* and *Bacillus cereus* clades are not phylogenetically related, the *Bacillus* genus is restricted only to members of these two clades. The *subtilis* clade is actually *Bacillus sensu stricto*, as it contains the type strain *Bacillus subtilis.* On the other hand, however, the *cereus* clade cannot be named a separate genus as it contains multiple human pathogens according to Rule 56a of the International Code of Nomenclature of Prokaryotes (Gupta et al., 2020; Oren et al., 2023). *Bacillus cereus* group members often contaminate food and feedstock, as their spores can survive dehydration and pasteurization processes. Distinguishing markers between three pathogenic *Bacillus cereus* group members (*Bacillus cereus*, *Bacillus thuringiensis*, *Bacillus anthracis*) are often encoded by genes contained in plasmids. *Bacillus cereus* is an opportunistic pathogen that causes food poisoning to which chromosomally encoded Nhe (non-hemolytic enterotoxin), Hbl (hemolytic enterotoxin), and CytK (cytotoxin K) protein toxins contribute, as well as cereulide toxin on its megaplasmid pCER270 (Ehling-Schulz et al., 2019). *Bacillus thuringiensis* is known for its insecticidal activity due to the presence of *cry*, *cyt* and *vip* genes (*Bt* genes) on its large plasmids; however, biopesticides based on *B. thuringiensis* are recognized as safe for humans (Ehling-Schulz et al., 2019; Raymond and Federici, 2017). *Bacillus anthracis* holds historical significance in microbiology as it was the first bacterium discovered to be a pathogen and the first evidence for the germ theory of diseases (Blevins and Bronze, 2010). Its pathogenicity depends on two plasmids, pXO1 and pXO2, containing genes encoding for the anthrax toxin (*pag*, *lef*, *cya*) and polyglutamate capsule (*capABCDE*) (Blevins and Bronze, 2010; Koehler, 2002).

**FIGURE 2:**
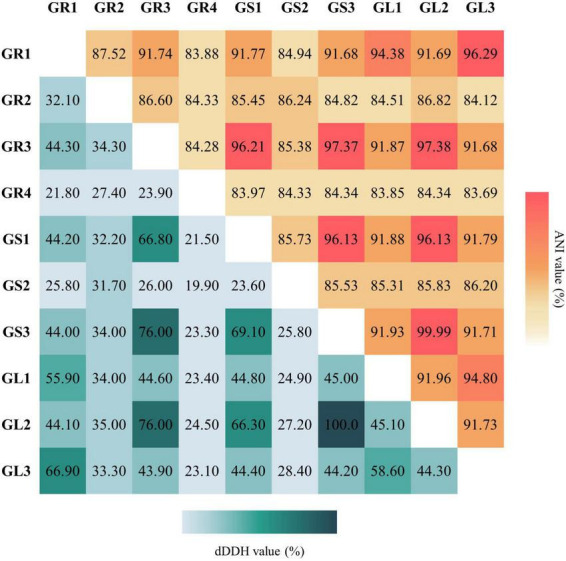
Average Nucleotide Identity (ANI) and digital DNA:DNA hybridization (dDDH) values between isolated bacterial endophytes’ genomes. ANI (%; ANIm results) values are indicated in the upper triangle and dDDH (%; formula d4 results) in the lower triangle.

To validate the taxonomic identification of *Bacillus cereus* group members, the BTyper3 tool was used (Carroll et al., 2020). The identification of the strains presented in Table 2 was confirmed, and they were assigned to two different phylogenetic groups based on *panC* sequence (pantoate-beta-alanine ligase gene). GR3, GL2, GS1, and GS3 were assigned to group IV (*Bacillus cereus* sensu stricto), while GR1, GL1, and GL3 strains were assigned to group II (*Bacillus mosaicus*/*luti*). Neither anthrax nor emetic toxin cereulide encoding gene was found in any genome; all of them contain *nhe* and *hbl* gene clusters and *sph* (sphingomyelinase C) gene. The *cytK* gene was identified only in group IV members (*Bacillus cereus* sensu stricto). No genes encoding *Bt* proteins, including the GS1 genome, were detected. Still, strain GS1 is identified as *Bacillus thuringiensis* due to higher % of ANI and dDDH values between *Bacillus thuringiensis* ATCC 10,792 than *Bacillus cereus* ATCC 14,579. No putative circular plasmid sequence has been detected in GS1 genome; however, there are known cases of *B. thuringiensis* without its characteristic plasmids (Bianco et al., 2021). Strains GR3, GL2, and GS3 were classified as *Bacillus cereus*. Comparison between strains (Figure 2) suggests that GS3 and GL2 may be the same strain, which putatively translocated through the stem to the leaf, with 100% and 99.99% as ANI and DDH values, respectively. Strains GL3 and GR1 were classified as *Bacillus wiedmannii* and *Bacillus pretiosus*, respectively, and GL1 as *Bacillus* sp. with close relation to them. Interestingly, strain GR1 is only the second example of *Bacillus pretiosus*; the first one being isolated from the rhizosphere of *Medicago sativa* in 2022 (Robas Mora et al., 2023).

Researchers have investigated few medicinal plants for the presence of beneficial endophytes, most of which contain *Bacillus*-related species with multiple plant growth-promoting activities. For instance, 65% of bacteria isolated from licorice (*Glycyrrhiza uralensis* F.) were found to belong to the *Bacillus* spp. based on 16S rDNA sequences, and most of them were able to produce indole-3-acetic acid (IAA) and siderophores, fix nitrogen, and solubilize phosphate (Li et al., 2018). Marchut-Mikołajczyk et al. (2023) isolated two *Bacillus cereus* strains and one *Bacillus mycoides* with the capability of producing polyphenols present in their host plant, *Urtica dioica*. The medicinal plant *Alectra sessiliflora* comprises five endophytic strains from *Lysinibacillus*, *Peribacillus*, and *Bacillus* genera that exhibit notable antibacterial and antitumor activities (Maela et al., 2022). Nisa et al. (2022) managed to obtain all three most iconic strains of *Bacillus cereus* group – *Bacillus cereus*, *Bacillus thuringiensis* and *Bacillus anthracis* - from the medicinal plant *Berberis lycium*; however, identification was based solely on 16S rDNA sequence. Four strains inhabiting *Origanum vulgare* L. were found capable of growing on diesel fuel, probably because it resembled the hydrocarbon components of its essential oil, and all of them belong to *Bacillus* genera based on their whole genome sequences – two *Priestia megaterium*, *Metabacillus dongyingensis*, and *Paenibacillus xylanexedens* (Semenzato et al., 2022b).

Also, *Bacillus*-related bacterial species are often identified as the main endophytic species enhancing plant stress tolerance in extremophilic conditions, which emphasizes the importance of the plant microbiome for its survival and fitness. Bokhari et al. (2019) examined the potential of *Bacillus* strains isolated from five desert medicinal plants. Only under salt stress conditions, *Bacillus cereus*, *B. subtilis*, and *B. circulans* isolates significantly enhanced growth of non-host plants (Bokhari et al., 2019). *Priestia* spp. are also known as very advantageous endophytes. Its prime example, *Priestia megaterium*, has been found to control pear fire blight disease caused by plant pathogen *Erwinia amylovora* (Cui et al., 2023). Similarly, researchers have recognized *Priestia aryabhattai* from wheat as a multi-stress reducer (Shahid et al., 2022). The potential for biofertilization with *Bacillus* endophytes is also well-known (Etesami et al., 2023). Recently, co-inoculation of rice, an important crop in the food industry, with endophytic *B. siamensis* and *P. megaterium*, was found to significantly improve its growth (Rios-Ruiz et al., 2023). In addition, pre-sowing treatment of wheat seeds with endophytic *B. subtilis* and salicylic acid reduced drastically the development of *Fusarium culmorum* instigated root disease in mature plants while simultaneously increasing yields under normal and drought conditions (Lastochkina et al., 2020).

Numerous novel *Bacillus*-related strains are of endophytic origin, including for example *Bacillus endophyticus* (2002), *Bacillus graminis* (2011), *Bacillus endoradicis* (2012), *Bacillus lycopersici* (2015), *Bacillus cabrialesi* (2019), *Bacillus taxi* (2020), *Bacillus mexicanus* (2023), *Bacillus dicomae* (2023) (Bibi et al., 2011; de los Santos Villalobos et al., 2019, 2023; Lin et al., 2015; Makuwa et al., 2023; Reva et al., 2002; Tuo et al., 2020; Zhang et al., 2012), all published validly in *International Journal of Systematic and Evolutionary Microbiology* by the Microbiology Society. However, still not so many whole genomic sequences of endophytic bacteria have been deposited in the NCBI database, leaving open doors for insightful analyses of the features responsible for the wide distribution of *Bacillus*-related bacteria as endophytes.

## Functional annotation

### Genes associated with plant growth promotion

**FIGURE 3:**
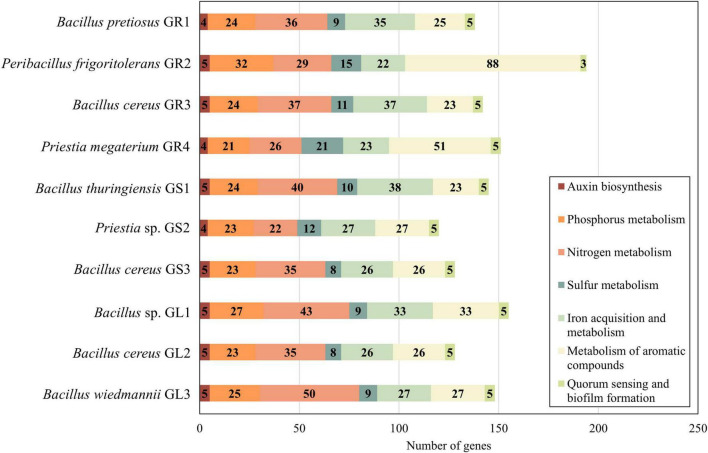
Presence of genes associated with plant growth promotion according to SEED functions from The Rapid Annotation using Subsystem Technology (RAST).

A substantial amount of genes is significant to the formation and maintenance of an endophytic lifestyle, as well as indirectly for plant growth promotion, adaptation and protection (Figure 3).

Endophytic bacteria enhance the metabolism of essential nutrients in their plant hosts, such as phosphorus, nitrogen, and sulfur, mostly by increasing the bioavailability of these elements, which later serve for amino acids and protein synthesis (Chaudhary et al., 2022). *Galium aparine* L.’s bacteria possess genes encoding various phosphatases (EC 3.1.3.1, EC 3.6.1.11, EC 3.1.3.18, etc.) which catalyze solubilization of insoluble phosphorus forms so the plant can absorb it or mobilize its uptake by binding proteins (e.g., PhnK, PhnL) facilitating the transport onto the roots (Walia et al., 2017). In regards to nitrogen, few acquisition mechanisms are possible, however, in the studied strains, there is a high predominance of enzymes associated with ammonia assimilation (glutamate synthase – EC 1.4.1.13, glutamine synthetase – EC 6.3.1.2), while at the same time, all genomes lack nitrogen fixation enzymes. Additionally, these endophytes can also mineralize organic sulfur compounds in the soil and secrete enzymes such as alkanesulfonate monooxygenase (EC 1.14.14.5), thioredoxin reductases (EC 1.8.1.9) and peroxidases (EC 1.11.1.15) to convert sulfates into sulfites that can be assimilated by plants. Iron acquisition and metabolism category comprises mostly genes related to siderophores synthesis and iron transport. Iron is a crucial micronutrient for plants as it is involved in chlorophyll synthesis, and further maintenance of chloroplast structure, not to mention that it is required by many enzymes for proper work (Rout and Sahoo, 2015). Quorum sensing and biofilm genes do not vary across strains, they contain gene for S-ribosylhomocysteine lyase (EC 4.4.1.21) and S-adenosylmethionine synthetase (EC 2.5.1.6), which take part in the formation of an autoinducer (Al-2) in the quorum-sensing mechanism. Metabolism of aromatic compounds is important to environmental strains as they often have to provide themselves with nitrogen and carbon sources from recalcitrant waste substrates and/or toxic aromatic compounds. Except for *Peribacillus frigoritolerans* GR2, studied endophytes exhibit similar repertoires of enzymes involved in n-phenylalkaloic acid, phenylpropanoid, quinate, gentisare degradation, indicative of their shared environmental origin.

A pivotal plant growth-promoting trait of many bacterial endophytes is their ability to synthesize indole-3-acetic acid (IAA), primarily by tryptophan-dependent pathway (Tang et al., 2023). As a member of the auxin family of indole derivatives, IAA regulates almost every aspect of plant development, including cell division, roots and stem elongation, as well as responses to environmental cues (Duca and Glick, 2020; Tang et al., 2023). Strains *Bacillus pretiosus* GR1, *Peribacillus frigoritolerans* GR2, *Priestia megaterium* GR4, and *Priestia* sp. GS2 possess genes involved in tryptophan biosynthesis, including anthranilate phosphoribosyltransferase (EC 2.4.2.18), phosphoribosylanthranilate isomerase (EC 5.3.1.24), tryptophan synthase alpha chain (EC 4.2.1.20) and tryptophan synthase beta chain (EC 4.2.1.20). The rest of the strains (all belonging to *Bacillus* genus) additionally have indole-3-pyruvate decarboxylase (EC 4.1.1.74), which facilitates the conversion of indole pyruvate (IPA) to indole-3-acetaldehyde (IAAld), suggesting that most likely the IAA production occurs according to the IPA pathway, which was first discovered in plants, now one of the major pathways for microbial IAA biosynthesis (Tang et al., 2023).

While a single endophytic species alone is not able to drastically improve plant fitness and life conditions, a whole group of them can significantly contribute. Notably, certain species demonstrate “specialization” in specific plant growth-promoting traits, as exemplified by *Peribacillus frigoritolerans* GR2, which possesses almost two times more enzymes connected to degradation (especially n-phenylalkanoic acids) than *Bacillus wiedmannii* GL3 with its heightened amount of reductatases for organic sulfur mineralization and assimilation. Given their well-studied plant growth-enhancing properties, *Bacillus* spp. stand out as exemplars for future research on the cruciality of endophytes for plant metabolism and the development of sustainable agriculture agents.

### Mobile genetic elements (MGEs)

As major drivers of horizontal gene transfer (HGT), bacterial mobile genetic elements (MGEs), also known as the “bacterial mobilome,” can move within the genome or transfer between bacterial species, ensuring their better adaptation to the occupied ecological niche. That broad term is represented not only by plasmids and phages but also by transposable elements, insertion, restriction and modification systems, integrative and conjugative elements (ICEs) among others (Khedkar et al., 2022).

Five different categories of MGEs provided by mobileOG-db database include integration and excision (IE; recombinases, transposases, etc.) replication, recombination or nucleic acid repair (RRR; repair and recombination systems, plasmid and phage replication initiators, etc.), phage-related biological processes (P; lysis and lysogeny-associated machinery, etc.) stability, transfer, or defense (STD; CRISPR proteins, etc.) and inter-organism transfer (T; conjugation machinery, etc.); each one corresponding to another key molecular machinery (Brown et al., 2022). Table 3 shows that *Bacillus* spp. strains (GR1, GR3, GS1, GS3, GL1, GL2, GL3) have approximately 180–200 mobile genetic elements (MGEs), whereas *Peribacillus frigoritolerans* GR2, *Priestia megaterium* GR4, and *Priestia* sp. GS2 has slightly fewer, with *Priestia* sp. GS2 has just 94 MGEs.

**TABLE 3:** Mobile genetic elements (MGEs) assigned to major mobileOG categories.

MGEs category	GR1	GR2	GR3	GR4	GS1	GS2	GS3	GL1	GL2	GL3
Integration/excision (IE)*	51	40	70	24	68	14	61	73	61	86
Replication/recombination/repair (RRR)*	52	44	47	57	50	42	46	52	46	50
Phage (P)*	44	16	32	23	48	18	39	33	38	29
Stability/transfer/defense (STD)*	9	4	7	6	6	6	13	6	12	9
Transfer (T)*	27	16	35	29	30	14	28	34	28	32
All	183	120	191	139	202	94	187	198	185	206

*IE, integration/excision; RRR, replication/recombination/repair; P, phage; STD, stability/transfer/defense; T, transfer.

These data suggest that the bacteria in question are not an exception in nature, as the movement of genetic material acts as a driving force in their adaptation and fitness by facilitating gene loss or gain, in further perspective contributing to evolution. The study of HGT in the context of endophytes and plants has been limited. However, accurate detection of its presence is essential for understanding the complex relationship between them and their mutual influences. This knowledge can directly help to enhance the application of bacteria in sustainable agriculture (Rajabal et al., 2024; Tiwari and Bae, 2020).

In an evolutionary context, a prime example of MGEs is bacteria’s acquisition of antibiotic resistance. The rise of antibiotic resistance is one of the most pressing problems in the modern world. According to the World Health Organization (WHO), 700,000 people die each year as a result of this phenomenon and that number is projected to increase to 10,000,000 by 2050 if no effective solutions are developed (Mancuso et al., 2021). The extensive use of antibiotics in clinical settings, hospitals, farm animals, agriculture, and aquacultures has created selective pressure in bacteria toward the acquisition and further dissemination of antibiotic-resistance genes among bacteria (Zhuang et al., 2021). Research conducted by Xu et al. (2021) showed that endophytic bacteria can easily spread antibiotic resistance genes (ARGs) carried on a plasmid.

The resistomes of isolated strains were analyzed to obtain information related to antibiotic resistance genes, which resulted in identification of five antimicrobial resistance (AMR) gene families, each displaying four resistance mechanisms (Table 4).

**TABLE 4:** Antimicrobial resistance (AMR) gene families predicted in *Galium aparine* L’s bacterial endophytes.

AMR* gene family	ARO* term	Drug class	Antibiotic resistance mechanism	Number of hits									
				GR1	GR2	GR3	GR4	GS1	GS2	GS3	GL1	GL2	GL3
Glycopeptide resistance gene cluster	*van*R/*van*T/*van*W/*van*Y	Glycopeptide antibiotic	Target alteration	8	3	11	6	10	4	9	8	9	9
*Bacillus cereus* Bc beta-lactamase	BcI/BcII/BcIII	Cephalosporin, penem	Inactivation	4	1	4	1	4	1	2	5	2	5
Fosfomycin thiol transferase	FosB	Phosphonic acid antibiotic	Inactivation	1	1	1	1	1	1	1	1	2	1
SMR* antibiotic efflux pump	*qac*J/*qac*G	Disinfecting agents and antiseptics	Efflux	1	2	1	3	1	1	1	1	1	1
Tetracycline-resistant ribosomal protection protein	tetB	Tetracycline antibiotics	Target protection	1	0	1	1	1	0	1	1	1	1
All				15	7	18	12	18	7	14	17	14	18

*ARO, antibiotic resistance ontology; AMR, antimicrobial resistance; SMR, small multidrug resistance.

Strains belonging to *Bacillus* spp. species (GR1, GR3, GS1, GS3, GL1, GL2, GL3) exhibit a comparable number and distribution of AMR genes, suggesting similar resistance to antibiotics among them. Conversely, strains from *Priestia* (GS2, GR4) and *Peribacillus* (GR2) genera have significantly lower numbers of AMR genes. The major family in all bacterial genomes encodes the glycopeptide antibiotic resistance proteins (*van*W, *van*Y, *van*R and *van*T genotypes), which function through the antibiotic inactivation resistance mechanism. Glycopeptide antibiotics, such as vancomycin and teicoplanin, are commonly used to treat life-threatening community-acquired infections caused by methicillin-resistant *Staphylococcus aureus* (MRSA) (Binda et al., 2014). In addition, *Bacillus cereus* Bc β-lactamase gene family was identified, which is implicated in carbapenems resistance (e.g., cephalosporin and β-lactams) by inactivation mechanism. Carbapenem-resistant bacterial strains are being detected in farm animals, due to increased use of antibiotics, and they are mostly plasmid-borne; thus, easily spread (Zhuang et al., 2021). The same resistance mechanism is adapted by fosfomycin thiol transferase encoded by *Fos*B gene family acting on phosphonic acid antibiotics, such as fosfomycin (acute cystitis treatment for pregnant women) and fosmidomycin (anti-malarial) (Chekan et al., 2016). Another AMR gene family acting via antibiotic efflux (impacting disinfecting agents and antiseptics) is primarily represented by *qac*J genotype; however, *qac*G is also additionally present in *Priestia megaterium* GR4, which confers resistance to quaternary ammonium compounds, like acriflavine, ethidium bromide, benzalkonium chloride, cetrimide, dequalinium (Jaglic and Cervinkova, 2012). Of all the strains, only *Peribacillus frigoritolerans* GR2 and *Priestia* sp. GS2 lack the *tetB* gene encoding for tetracycline antibiotic resistance. Tetracycline antibiotics, including tetracycline and oxytetracycline, are widely employed in veterinary medicine (Zhuang et al., 2021).

One of the other key factors shaping the ecological balance and evolution of bacteria are phages, the most abundant biological entities on Earth. As predators, they kill bacterial hosts by lysis, causing selective pressure for bacteria resistant to phage infection, which further results in the emergence of different adaptation systems, such as the CRISPR-Cas immune system, leading to co-evolution of phages and bacteria (Casas and Maloy, 2018; Grose et al., 2014). However, phages serve additional functions beyond predation. Phages have the ability to package fragments of any bacterial genome region into their capsids, which they then transfer to other bacterial hosts for integration into the genome through recombination (Chiang et al., 2019). This process, known as transduction, may confer additional advantages for occupied niches as AMR, virulence or novel metabolic properties, which can be fixed in the population. Also, phages can integrate into the bacterial genome during the lysogenic cycle, replicating within bacteria and, when induced, shifting to the lytic phase (Chiang et al., 2019). During dormancy, bacteria are often immune to related phages, yet too high energetic costs associated with maintaining the phage genes may force them to reduce their number. The persistence of their remnants within the genome raises inquiries regarding the potential fitness advantages they may confer; however, the precise nature of these benefits remains unclear (Casas and Maloy, 2018; Nedialkova et al., 2016). After identification of the genes and/or remnants of phages by MGEs analysis, whole phage regions were detected (Table 5).

**TABLE 5:** Phages identified in endophytic genomes.

Strain	Identified phage (GenBank Accession no.)	Region length (kbp)	Start	End	No. of total proteins	GC (%)
*Bacillus pretiosus* GR1	*Bacillus phage* phIS3501 (NC_019502)	58.5	3,173,596	3,232,186	68	36.78
	*Bacillus phage* IEBH (NC_011167)	50.8	779	51,609	90	36.40
*Bacillus cereus* GR3	*Bacillus phage* phi4J1 (NC_029008)	29.5	2,859,647	2,889,205	42	36.47
	*Brevibacillus phage* Jenst (NC_028805)	65.0	565730	630,753	59	33.45
*Priestia megaterium* GR4	*Bacillus phage* PM1 (NC_020833)	71.5	786,539	858,040	88	37.05
*Bacillus thuringiensis* GS1	*Geobacillus phage* GBSV1 (NC_008376)	58.3	671,230	725,088	50	34.83
	*Bacillus phage* phIS3501 (NC_019502)	41.0	1	41,007	61	35.17
*Bacillus cereus* GS3	*Bacillus phage* Vb_BhaS_171 (NC_03090)	19.9	4,150	24,136	32	35.86

As a result, the strains *Bacillus pretiosus* GR1, *Bacillus cereus* GR3 and *Bacillus thuringiensis* GS1 were found to harbor two copies of complete phage regions across their genomes, while *Priestia megaterium* GR4 and *Bacillus cereus* GS3 each contained one. *Bacillus* spp. are highly ubiquitous in many ecological niches, making them ideal subjects for studying host-phage interactions (Grose et al., 2014). The majority of the bacteriophage genes are associated with transcriptional regulation and phage machinery, such as capsid, tail, portal, terminase, integrase and replication proteins. While bacteriophages have been identified in some of the endophytic bacteria, their role in the adaptation to endophytic lifestyle and phytobeneficial traits has yet to be elucidated (Li et al., 2021; Wang, 2024). As mentioned before, clustered regularly interspaced short palindromic repeats (CRISPR)/CRISPR-associated (Cas) is a bacterial immunity mechanism based on the bacterial ability to incorporate a fragment of phage genome into their own as a spacer sequence between repeated sequences in CRISPR. When genetic material complementary to the spacer is detected, indicating phage infection, the system initiates degradation, protecting the bacterium (Watson et al., 2021). We identified CRISPR-Cas system elements in genomes of all endophytic bacteria isolates from *Galium aparine* L. (Supplementary Table 1).

Lastly, we searched for putative plasmid sequences within our genomic data, as plasmids represent the largest and most commonly encountered examples of MGEs. First, we used PlasmidSPAdes to find plasmid sequences from short-reads (Antipov et al., 2016). Then we compared these results with circular contigs that we got from Unicycler hybrid assemblies. These sequences were then subjected to a BLASTN search against the NCBI Nucleotide database, and those showing hits to plasmids were further analyzed using the PLSDB database (Schmartz et al., 2022). Results are shown in Table 6. We subjected the identified putative plasmid sequences to Prokka annotation, searched for MGEs, ARGs, and phages, and then visualized as circular map (Supplementary Figure 1).

**TABLE 6:** Putative plasmid sequences in *Galium aparine* L’s bacterial endophytes.

Strain	Contig no.	Length (kbp)	GC (%)	CDSs*	MGEs*	ARGs*	CRISPR/Cas*	PLSDB (NCBI accession no.)		
								Best match	Cover (%)	Identity (%)
*Bacillus pretiosus* GR1	2	383.181	32.59	365	43	1	0	*Bacillus wiedmannii* bv. *thuringiensis* strain FCC41 plasmid pFCC41-1-490K (CP024685.1)	62	99.48
	5	54.381	36.3	94	15	0	0	–	–	–
*Bacillus cereus* GR3	3	637.249	32.03	579	71	2	0	*Bacillus thuringiensis* strain B13 plasmid pBt13367-1 (NZ_CP074713.1)	41	98.93
*Priestia megaterium* GR4	22	7.738	35.38	8	3	0	0	*Priestia megaterium* QM B1551 plasmid pBM200 (NC_010009.2)	79	92.51
*Bacillus* sp. GL1	4	213.927	32.83	188	26	1	1	*Bacillus tropicus* strain EMB20 plasmid pBEMB20-2 (NZ_CP078083.1)	47	96.56
	6	72.347	31.66	96	11	0	0	*Bacillus mycoides* strain BPN43/2 plasmid p75 (NZ_CP036011.1)	74	90.88
	9	3.965	34.91	4	1	0	0	*Bacillus mycoides* plasmid pBMY1 (NC_005703.1)	55	86.41
*Bacillus wiedmannii* GL3	2	464.727	32.40	409	41	2	0	*Bacillus wiedmannii* strain EPS29 plasmid ppl557 (NZ_CP133558.1)	70	99.49
	3	226.263	32.72	231	30	0	0	*Bacillus thuringiensis* HD-771 plasmid p02 (NC_018501.1)	44	89.89
	4	6.402	30.55	7	2	0	0	–	–	–

*CDSs, coding sequences; MGEs, mobile genetic elements; ARGs, antibiotic resistance genes; CRISPR/Cas, clustered regularly interspaced short palindromic repeats (CRISPR)/CRISPR-associated (Cas).

Half of the isolated strains contain putative plasmid sequences, some even more than one. Except for two hypothetical plasmids (*Bacillus pretiosus* GR1, contig 5; *Bacillus wiedmannii* GL3, contig 4), all of them were matched to plasmids present in the PLSDB database; additionally, all even to those from taxonomically matching strains’ genera. Putative plasmid sequences have lower GC content than whole genomes (Table 1), and some of them are especially large (up to 10% of the whole genome as with *Bacillus cereus* GR3), which makes them so-called megaplasmids. Thresholds for minimum megaplasmid size vary as the overall genome size should be considered: however, ≥ 350 kbp proposed by DiCenzo and Finan (2017) based on 10% of the median bacterial genome size is generally accepted cut-off. Here, we report three examples of such putative plasmids (Table 6), all encoding a plethora of enzymes useful for the occupied niche, e.g., esterases, peptidoglycan-N-acetylglucosamine deacetylases, bacillolysin, and biosynthetic gene clusters, which were manually found using BLASTN in various other *Bacillus* plasmids. Thanks to the growing availability of short- and long-read sequencing, which enables hybrid assembly and high-quality genome, it is possible to detect not only plasmids and megaplasmids, but also other more peculiar replicons, such as secondary chromosomes and chromids. The main difference is that chromids (originating from megaplasmids) and secondary chromosomes (from a split of an ancestral chromosome) carry essential housekeeping genes found on the chromosome in other species, which is often confirmed experimentally (Hall et al., 2022).

None of the identified plasmids were similar to those encoding toxins characteristic of *Bacillus anthracis* or *thuringiensis* from *Bacillus cereus* group, which further confirms that they are not members of those genera. However, there is a high possibility that strain GS1 is a member of *Bacillus thuringiensis*, but has lost its characteristic plasmid, making it difficult to differentiate from *Bacillus cereus*. Conversely, strain GR3 classified as *Bacillus cereus* member based on ANI and dDDH parameters surpassing thresholds in comparison to the type strain, does possess *Bacillus thuringiensis* plasmid, although it lacks *cry* gene encoding insecticidal crystal protein. In addition, the best match according to the PLSDB database is *Bacillus thuringiensis* strain B13 plasmid pBt13367-1, which also does not possess that gene. Interestingly, *cry* gene was found in the chromosome sequence within a genomic island, the same as in *Bacillus thuringiensis* HER1410 genome (Lechuga et al., 2020; Shwed et al., 2022). These findings highlight the significance and complexity of horizontal gene transfer events in bacteria, showcasing the permanent integration of plasmid-encoded traits into the chromosome structure.

### Carbohydrate-active enzymes (CAZymes)

Carbohydrate-active enzymes are families of enzymes intricately associated with the synthesis, degradation and modification of carbohydrates, divided into five classes based on their function within the CAZy database^1^. These classes comprise glycoside hydrolases (GHs), carbohydrate esterases (CEs), glycosyl transferases (GTs), polysaccharide lyases (PLs), and now also non-catalytic carbohydrate-binding modules (CBMs) and auxiliary activity enzymes (AAs) acting in collaboration with other CAZymes to enhance their activity (Davies et al., 2018).

**FIGURE 4:**
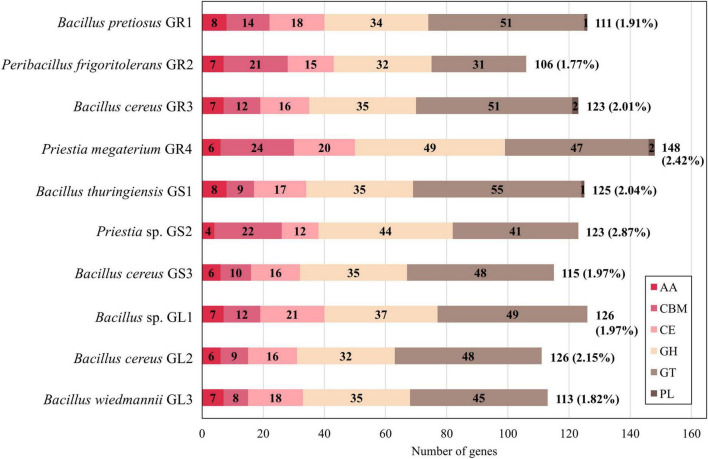
Comparison of the distribution of carbohydrate-active enzyme (CAZyme) classes identified in *Galium aparine* L.’s bacterial endophytes. AA, auxiliary activity; CBM, carbohydrate-binding module; CE, carbohydrate esterase; GH, glycoside hydrolase; GT, glycosyltransferase; PL, polysaccharide lyase.

As a result of CAZymes identification analysis, between 106 (*Peribacillus frigoritolerans* GR2) and 148 (*Priestia megaterium* GR4) genes were mapped to the CAZymes family. Figure 4 illustrates the distribution of predicted CAZymes’ families across the genomes. The number behind the bar indicates the total number of CAZymes, while the number in brackets represents the percentage of CAZymes to the total number of genes predicted by RAST.

The proportions of CAZymes families differ slightly depending on the species represented. It was the glycosyl transferases (GTs) that were most common in *Bacillus* spp. strains, followed by glycoside hydrolases (GHs) and then by carbohydrate esterases (CEs) and carbohydrate-binding modules (CBMs). In comparison, in strains belonging to *Priestia* spp. and *Peribacillus* spp., the amount of GTs and GHs is similar and CBMs are more abundant than in *Bacillus* spp. CEs and AAs levels are similar in all strains. Comprehensive analysis showed the highest abundance of CBM50 (LysM domains binding to the N-acetylglucosamine residues in bacterial peptidoglycan and chitin), CE4 (esterases catalyzing the de-acylation of polysaccharides), GH13 (hydrolases acting on substrates with α-glucoside linkages), GT2 and GT4 (catalyzing glycoside synthesis) families (CAZypedia, 2024; Davies et al., 2018 and Supplementary Figure 2). Interestingly, only *Bacillus cereus* GR3 and *Priestia megaterium* GR4 contain enzymes from PL12 family (heparin lyases activity) (Cazy, 2024). PL9 family is more frequent across strains (GR1, GR3, GR4, GS1); its activity is connected to pectin cleaving, a major plant cell wall polysaccharide (Cazy, 2024).

We have used the number, type, and proportions of CAZymes an organism carries as a marker to assess its adaptation to a specific environment and gain insights into its lifestyle. Polysaccharides consist of diverse glycosyl units, often branched. Microorganisms can use these polysaccharides as sources of carbon and energy, acting as an adaptation mechanism to deal with temporary periods of starvation. The glucose units have to be released by specific enzymes – the key ones belonging to GHs (Wang et al., 2023). GHs and GTs are the most abundant across the genomes of many plant growth-promoting bacteria (PGPB) (Wang et al., 2023). GTs synthesize extracellular polysaccharides, which are crucial also for biofilm formation, resistance to environmental pressures, and other significant activities for endophytic bacteria (Wang et al., 2023). In *Bacillus* spp. strains there is no significant difference in the number and distribution of CAZymes between soil-, leaf- and other-associated PGPB, which is contrary to other common PGPB species (e.g., *Pseudomonas*, *Burkholderia*); thus, makes *Bacillus* spp. remarkably stable genus (Wang et al., 2023). Also, endophytic isolates from *Galium aparine* L. displayed a proportion of CAZymes to all predicted gene sequences ranging from 1.77% in *Peribacillus frigoritolerans* GR2 to 2.87% in *Priestia* sp. GS2 (Figure 4). Free-living organisms typically exhibit a CAZyme repertoire ranging from 1% to 5% of all predicted sequences. A significant reduction suggests a strict intracellular parasitic lifestyle (Lombard et al., 2014). Endophytic bacteria can not only colonize plants through natural openings and wounds, but some of them can do it actively using cell wall-degrading enzymes (cellulases, xylanases, etc.). *In planta*, the production of such hydrolases can help the plants establish systemic resistance against various pathogenic attacks, particularly chitinases and cellulases, as their activities correlate with the biocontrol of fungal plant pathogens (Xu et al., 2020).

Bacterial CAZymes are successfully applied for multiple biotechnological (e.g., food processing, detergent additives), medical (e.g., synthesis of pharmaceutical intermediates) and industrial (e.g., xenobiotics degradation, dye production) purposes as they are often more sustainable, cheaper and time efficient solution. Genome mining for CAZymes is a very useful tool for picking the right bacteria and planning more experiments to break down tough substrates like cellulose, starch, lignin, and others. Because those substrates possess unique complex structures, enzymes have to work in conjunction for that purpose. For instance, for effective and complete degradation of chitin, the polymer of (1→4)-β-linked N-acetyl-D-glucosamine (GlcNAc), and chitosan, its deacetylated form, not only chitinases and chitosanases are needed, but also deacetylases, aminidases and lytic monooxygenases (Kaczmarek et al., 2019). Endophytic bacteria are valuable sources of biotechnologically important enzymes; however, still little information is available on their abundance and potential.

**FIGURE 5:**
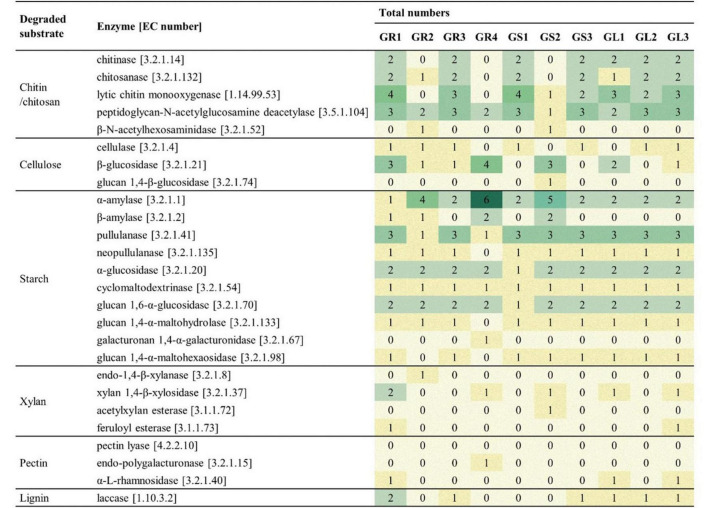
Genes associated with polysaccharide degradation.

The CAZyme repertoires from *Galium aparine* L. endophytes were manually screened for the presence of specific enzymes associated with the degradation of selected polysaccharides (Figure 5).

All isolated strains display genomic potential for the degradation of polysaccharides, especially chitin, chitosan, cellulose and starch. Strains belonging to *Bacillus* spp. demonstrate a higher number of genes encoding enzymes associated with chitin and chitosan degradation, while strains from *Priestia* spp. those associated with starch hydrolysis, e.g., six copies of an α-amylase gene in *Priestia megaterium* GR4. Only a few genes associated with xylan, pectin and lignin degradation were found across the genomes of all isolates. However, the enzymes presented in Table 4 are only known enzymes with assigned EC number, other enzymes involved in the degradation process may be “hidden” on the family level.

### Biosynthetic gene clusters (BGCs)

Bacteria are capable of synthesizing diverse biologically active compounds during their secondary metabolism. Most of the secondary metabolites serve as traits bringing fitness advantages to the occupied ecological niche and microbial community, including cell-cell signaling or nutrient scavenging. However, their production also brings costs to the individual cell as it uses resources from primary metabolism (Santamaria et al., 2022). Apart from their ecological role, secondary metabolites have significant potential as therapeutics and agricultural agents. Traditional approaches for screening for their production by bacteria involved bioactivity-based assays of their culture supernatants, their extraction and fractionation with solvents and attempts at characterization by such techniques as mass spectroscopy (MS) and nuclear magnetic resonance spectroscopy (NMR). These processes are both expensive and labor-intensive and often lead to the rediscovery of known compounds. As a result of the greater affordability of DNA sequencing, genome mining approach has emerged. Through recognizing specific genetic sequences encoding highly conserved enzymes, biosynthetic gene clusters (BGCs) responsible for producing these metabolites can be identified in bacterial genomes (Russell and Truman, 2020; Yvens et al., 2022). BGCs are categorized based on the presence of such highly conserved enzymes; two main BGCs types are non-ribosomal peptide synthetases (NRPS) and polyketide synthases (PKS), which are produced solely by encoded enzymes, not by ribosomes (Yvens et al., 2022). Ribosomal products are the result of another BGCs type, coding for ribosomally synthesized and post-translationally modified peptides (RiPPs) (Russell and Truman, 2020).

**FIGURE 6:**
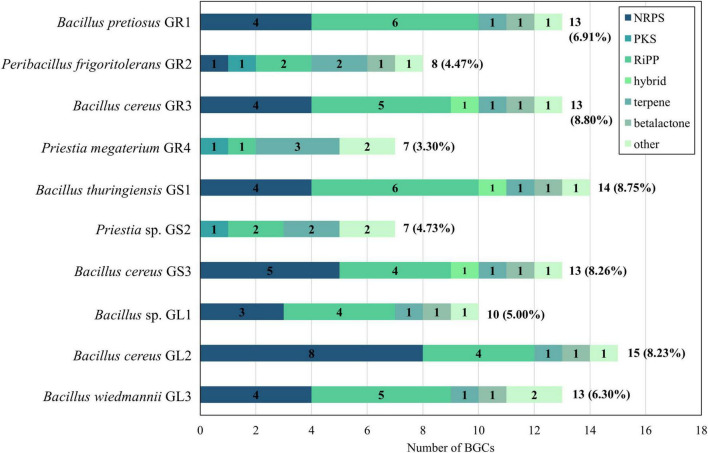
Comparison of the distribution of biosynthetic gene clusters (BGCs) types. NRPS, non-ribosomal peptide synthetase; PKS, polyketide synthase; RiPP, ribosomally synthesized and post-translationally modified peptides.

Genomes of *Galium aparine* L.’s bacterial endophytes were analyzed using the antibiotics and secondary metabolite analysis shell (antiSMASH) 7.0 tool to explore their secondary metabolites BGCs (Blin et al., 2023; Figure 6).

Bacteria dedicate approximately 4%–8% of their genomes to the production of secondary metabolites, with *Bacillus cereus* (GR3, GS3, GL2) and *Bacillus thuringiensis* (GS1) species reaching the upper limit of this range. Strains belonging to *Bacillus cereus* species, both *Bacillus cereus* and *thuringiensis* and *Bacillus wiedmannii* (GL1, GL3, GR1) subgroups, overall exhibit a higher number of clusters (10–15) compared to those in *Priestia* spp. and *Peribacillus frigoritolerans* GR2 (7–8).

The diversity of BGCs types also demonstrates a genus-specific distribution pattern. NRPS and RiPP clusters are more prevalent in *Bacillus* spp. strains (Grubbs et al., 2017; Yin et al., 2023 and Supplementary Table 2). Only one NRPS cluster in all *Bacillus* spp. strains display significant similarity to genes encoding known compounds in Minimum Information about a Biosynthetic Gene Cluster (MIBiG). That cluster is similar in 85% to bacillibactin from *Bacillus subtilis* subsp. *subtilis* str. 168 (MIBiG no.: 0000309), which is a well-described *Bacillus*-specific siderophore (Lalitha and Nithyapriya, 2021). A broad range of RiPPs, including lanthipeptides, sactipeptides and linear azol(in)e-containing peptides (LAP) was found, although only a few clusters showed ≥ 50% similarity to clusters encoding known compounds. An example is the sactipeptide cluster from *Bacillus cereus* GR3, which shares 70% gene similarity to thuricin H from *Bacillus thuringiensis* SF361 (MIBiG no. BGC0000600). Notably, these strains lack any PKS cluster. However, *Bacillus cereus* GR3 and *Bacillus thuringiensis* GS1 each possess one RiPP-NRPS-T1PKS hybrid cluster with 100% of genes similar to genes responsible for biosynthesis of zwittermicin A in *Bacillus cereus* UW85 (MIBiG no.: BGC 0001059). The presence of zwittermicin A is particularly intriguing in terms of plant-endophyte interaction as it exhibits various plant beneficial traits, such as antifungal activities, the ability to suppress plant disease caused by protists and the enhancement of the insecticidal activity of the toxins from *Bacillus thuringiensis* (He et al., 1994; Kevany et al., 2009). *Bacillus cereus* GS3 and *Bacillus cereus* GL2 both have NRPS-transAT-PKS hybrid cluster, though without significant gene similarity to known compounds. All of *Bacillus* spp. members possess one terpene and one beta-lactone gene cluster, as well as one in the “other” category, which includes NRPS-independent siderophore gene cluster similar to petrobactin from *Bacillus anthracis* str. Ames (100%, MIBiG no.: 0000942). In addition to the “other” category, *Bacillus wiedmannii* GL3 encodes a cluster on its putative plasmid sequence featuring a tRNA-dependent cyclodipeptide synthase (CDPS) as the core enzyme with 66% similarity to the pulcherriminic acid encoding cluster from *Bacillus subtilis* subsp. *subtilis* str. 168 (MIBiG no. BGC0002103).

Strains of *Priestia* spp., *Priestia* sp. GS2, *Priestia megaterium* GR4, and *Peribacillus frigoritolerans* GR2 contain fewer BGCs in comparison to *Bacillus* spp. members. They lack NRPS clusters but have a greater number of terpene gene clusters. Both *Priestia* spp. strains harbor a carotenoid gene cluster (terpene; 50% with *Halobacillus halophilus* DSM 2266; MIBiG no. BGC0000645) and type III PKS cluster. Interestingly, *Priestia* sp. GS2 possesses gene cluster encoding bacillopaline (other; 100% similarity with *Paenibacillus mucilaginosus* KNP414; MIBiG no. BGC0002488), metallophore known for its zinc-binding ability (Morey and Kehl-Fie, 2020). *Peribacillus frigoritolerans* GR2 contains an NRPS gene cluster with 87% similarity to the one encoding koranimine from *Bacillus* sp. NK2003 (MIBiG no. BGC0000377), which is also found in other endophytes of that genus (Maela and Serepa-Dlamini, 2023; Montecillo and Bae, 2022b).

As stated before, secondary metabolites are not essential for bacterial growth, but enhance their chances of survival in the occupied environment; a principle that applies also to endophytes. Many compounds, whose encoding clusters were found in *Galium aparine* L.’s endophytes, exhibit antibacterial and/or antifungal activities or metal-binding properties, directly influencing their ability to thrive inside the plant host. Although current knowledge about endophytic lifestyle is extensive, detailed comparative studies of BGCs in endophytic and non-endophytic bacterial genomes are needed to properly understand the importance of secondary metabolism in the establishment and maintenance of endophytism. Many of these clusters, especially those without any similarity to known compound genes remain silent, meaning they are not expressed under standard cultivation conditions (Zarins-Tutt et al., 2016). Therefore, finding a way to activate their synthesis, both by simply testing various cultivation conditions by One Strain Many Compounds (OSMAC) strategy or molecular biology techniques, is crucial for a comprehensive understanding of bacterial metabolism.

## Conclusion

To our knowledge, this is the first research of the endophytic community of the medicinal plant *Galium aparine* L., and one of the few that thoroughly analyzes genomes of endophytic bacteria from the same plant host. We isolated ten strains of bacterial strains from different tissues of *Galium aparine* L. and performed high-quality *de novo* assembly of their genomes using both short and long reads. Taxonomic identification based on whole genome sequences showed that all of them are members of the Bacillaceae family with *Priestia* sp. GS2 and *Bacillus* sp. GL1 potentially representing new species. Additionally, *Bacillus pretiosus* GR1 was identified as the second member of its genus. We examined the distribution of mobile genetic elements, including phages and plasmids, as well as their repertoires of carbohydrate-active enzymes and secondary metabolites. Our findings show the rich biosynthetic capacity among the endophytes, which may not only play an important role in adaptation to the endophytic lifestyle but, moreover, offer potential for diverse biotechnological applications. Although *Bacillus*-related strains are well-known as plant growth promoters, their full metabolic capacities are yet to be explored. Further detailed genome-guided studies are needed as they can lead to the discovery of novel enzymes and metabolites. Medicinal plants are a particularly rich and valuable source of bioactive compounds, yet their bacterial endophytes are still highly. Therefore, research focused on isolating culturable endophytes and exploring their biotechnological potential is of high importance.

## Materials and methods

### Isolation of endophytic bacteria

Endophytic bacteria were isolated from healthy *Galium aparine* L. herb collected in spring 2022 from a neighboring area of a military airport in Łask, Poland (51°34′02.8″N 19°11′04.6″E). The plant was dug up and quickly transported to the laboratory. After cleaning with tap water to remove soil particles, healthy parts were subjected to surface sterilization (90% ethanol - 3 min, 6.25% sodium hypochlorite – 5 min, 90% ethanol – 30 s), followed by rinsing five times in sterile water (Marchut-Mikolajczyk et al., 2018). Sterilized plant fragments, as well as 200 μl of water from last rinsing as sterilization control, were placed on LB agar medium (bactopeptone, 10 g; sodium chloride, 10 g; yeast extract, 5 g; agar, 25 g; dH_2_O, 1 L; pH 7.0 ± 0.2) and incubated for 7 days at 30°C. Obtained bacterial colonies were purified, Gram-stained and maintained in −80°C as 25% (v/v) glycerol stocks (Coico, 2005).

### DNA extraction, library preparation and sequencing

Genomic DNA of all strains was extracted from overnight cultures (LB medium, 30°C, 120 rpm) using both Gram Plus and Yeast Genomic DNA Purification Kit (Eurx, Poland), following the manufacturer protocol, as well as using phenol-chloroform method for Gram-positive bacteria (Wilson, 2001). Obtained genomic DNA was quantified with the Qubit dsDNA BR Assay Kit (Life Technologies), and its purity and integrity were assessed by spectrophotometric absorbance measurement and by agarose gel electrophoresis, respectively. For strains GS2, GS3, GL1, GL2, GR1, GR2, GR3 paired-end libraries (2 × 150 bp) were prepared from kit-extracted gDNA using MGIEasy FS PCR-Free DNA Library Prep Set and sequencing was performed at the BGI-TECH (Wuhan, China) on MGISEQ-2000 Sequencer (MGI, Shenzhen, China); for strains GS1, GL3 and GR4 TruSeq DNA PCR-Free Kit was used and sequencing was performed by Macrogen Europe on Illumina system sequencer. Phenol-chloroform extracted gDNA was subjected to library preparation by Native Barcoding Kit 24 V14 (Oxford Nanopore Technology) and NEBNext Companion Module (New England Biolabs) for subsequent sequencing on MinION Mk1b Nanopore sequencer (Oxford Nanopore Technology).

### Genome *de novo* assembly

Short reads underwent quality filtering and trimming using Trimmomatic v0.36 (Bolger et al., 2014). Nanopore-filtered reads were obtained after base calling, demultiplexing and debarcoding using Dorado v7.2.13 from Oxford Nanopore Technologies (ONT). FastQC v0.12.1 was used to examine the quality of reads (Andrews, 2010). *De novo* assembly of genomes was accomplished using Unicycler v0.5.1 using bold mode with an additional Pilon polishing step (Wick et al., 2017). PlasmidSPAdes v3.15.3 was used to assembly putative plasmid sequences from short-read data (Antipov et al., 2016) The assembly metrics and quality of obtained contigs was evaluated using QUAST v5.2.0 (Gurevich et al., 2013), while completeness and contamination with CheckM v1.2.2 (Parks et al., 2015) and BUSCO v5.5.0 (Manni et al., 2021).

### Taxonomic identification

The Type Strain Genome Server (TYGS) was used for initial whole genome-based taxonomic analysis against type-strain species present in their database and calculation of dDDH values (Meier-Kolthoff et al., 2022). The genomic data of the closely related strains were downloaded from the NCBI database, and further analyzed by JSpeciesWS tool to compute ANI values based on BLAST + (ANIb) and on MUMmer (ANIm) (Richter et al., 2016). Additionally, BTyper3 version 3.4.0 was used for further identification of *Bacillus cereus* group members, using multi-locus sequence typing (MLST), *panC* group assignment and virulence gene detection (Carroll et al., 2020). The Reference sequence Alignment based Phylogeny builder (REALPHY) tool was used for genome-wide comparisons of the closest related species, then MEGA v11.0.9 for the construction of a phylogenetic tree by the Neighbor-joining method with bootstrap values of 1,000 replications (Bertels et al., 2014; Tamura et al., 2021).

### Annotation and functional analysis

Genomes were annotated using NCBI PGAP (Tatusova et al., 2016) and RASTtk v1.073 (Aziz et al., 2008). Prediction of CRISPR and Cas proteins was done by the CRISPRCasFinder v4.2.20 (Couvin et al., 2018). The CARD v3.2.8 with RGI v6.0.3 was used to identify antibiotic resistance genes (perfect and strict hits-only mode) (Alcock et al., 2023). Phage regions were identified using the PHASTER tool (Arndt et al., 2016). The presence of bacterial MGEs was investigated by the program mobileOG-db 1.6v1 (Brown et al., 2022). Putative plasmid sequences were subjected to PLSDB database using mash dist mode, and their features were studied using the programs described above, and visualized using Proksee (Grant et al., 2023; Schmartz et al., 2022). CAZyme gene analyses were performed using the dbCAN3 server; searches were run against Pfam Hidden Markov Models (HMMs) database for sub- and family annotations (e-value < 1e-15, coverage > 0.35) with DIAMOND (e-value < 1e-102) for improved prediction accuracy (Zheng, 2023). Hits predicted by at least two tools were selected for analysis. Secondary metabolite gene clusters were found using bacterial antiSMASH v7.0 in relaxed detection strictness mode with all extra features on, including KnownClusterBlast, MIBiG cluster comparison, and transcription factor binding sequences (TFBS) analysis (Blin et al., 2023).

## Data Availability

The datasets presented in this study can be found in online repositories. The names of the repository/repositories and accession number(s) can be found below: https://www.ncbi.nlm.nih.gov/bioproject/?term=PRJNA1068863, PRJNA1068863.

## Footnotes

1
www.cazy.org
