# Supplementary material for: Exploring the diversity and genomics of cultivable Bacillus-related endophytic bacteria from the medicinal plant Galium aparine L
Source: Front Microbiol. 2025 Jun 30;16:1612860. doi: 10.3389/fmicb.2025.1612860 (PMC12256460; doi:10.3389/fmicb.2025.1612860)
Supplement: Supplementary file 1 [file Data_Sheet_1.pdf]

Table S1. CRISPR/CAS elements found in genomes of *Galium aparine* L.'s bacterial endophytes

Strain	Element	ID	Start	End	Spacer / Gene	Repeat consensus/cas genes	Direction
***Bacillus pretiosus* GR1**	CRISPR	contig01	12,428	12,533	1	CTCTTGATCCTCTCTTTTAA ACTTT	ND*
	CRISPR	contig01	853,202	853,436	3	TGTATGATTACCTTCCGCAT GAGAA	-
	CRISPR	contig01	1,383,762	1,383,888	1	CGTAGGGACGTTTTTTATTT AGGTCCTAACTTTGTTGT	ND
	CRISPR	contig01	2,857,780	2,857,880	1	CCTGTTGGTCCCCCAGGTG GACC	ND
	Cas cluster	contig03	80,773	82,359	2	cas3_TypeI, cas3_TypeI	-
***Peribacillus frigoritolerans* GR2**	CRISPR	Seq001	1,317,518	1,317,603	1	CAGAGTGGAGAGCTTCTTC AACAAATTCC	ND
	CRISPR	Seq001	1,398,974	1,399,161	3	CTGCTGGTGCGCCTTTGTG TGCTGCA	ND
	CRISPR	Seq001	4,298,226	4,298,310	1	GTATTTGCTCTGACTCACG AGTA	ND
***Bacillus cereus* GR3**	CRISPR	contig01	2,134,091	2,134,196	1	GGTCCAACTGGTGCAGAA GGTTCT	ND
	CRISPR	contig01	2,134,289	2,134,421	2	GGTCCAACTGGTGCAGAA GGTTCT	ND
	CRISPR	contig01	2,134,505	2,134,645	2	GGTCCAACTGGTGCAGAA GGTTCT	ND
	CRISPR	contig02	1,210,382	1,210,474	1	CCATCTACTAATTTTCCGAT ACCT	ND
***Priestia megaterium* GR4**	CRISPR	contig04	648,630	648,716	1	TAGTACGTCAAAAATGGCA AAAGAA	ND
	CRISPR	contig04	648,787	648,873	1	TAGTACGTCAAAAATGGCA AAAGAA	ND
	CRISPR	contig04	649,097	649,245	2	TAGTACGTCAAAAATGGCA AAAGAA	ND
	CRISPR	contig06	12,403	12,562	1	TTGTTTCGCTAGCAAATTC AGTACCGAAGTTGTTTTTA GATGCTTGAGCATTTTG	ND
	Cas cluster	contig15	14,585	16,105	2	cas3_TypeI, cas3_TypeI	-
	Cas cluster	contig19	9,342	10,742	2	cas3_TypeI, cas3_TypeI	-
***Bacillus thuringiensis* GS1**	CRISPR	contig03	683,247	683,342	1	TGGAGTTCTCAGAAAGAG CATTGT	ND
	CRISPR	contig05	213,971	214,094	1	AGAAGAAGTAACGGAAGA AGAAAAAGCGAAAGCCAA GGCG	ND
	CRISPR	contig05	230,560	230,693	2	GACAAATCTCAAAAAGAA GAGAA	+
	CRISPR	contig07	324,489	324,562	1	ATCATCATCATGGAGGACA CAAT	ND
	Cas cluster	contig12	8,853	10,442	1	cas3_TypeI, cas3_TypeI	-
	Cas cluster	contig14	24,319	27,849	2	cas3_TypeI, cas3_TypeI	-
***Priestia* sp. GS2**	CRISPR	contig01	3,175,544	3,175,752	3	TGTAAATGAGGTGTTTGTC AAAGA	ND
	CRISPR	contig02	12,049	12,154	1	CAGCTTGTGCGTTTTGTTG CTTCACGTGTTG	ND
	Cas cluster	contig04	32,753	34,261	2	cas3_TypeI, cas3_TypeI	-

***Bacillus cereus* GS3**	CRISPR	contig03	683,247	683,342	1	TGGAGTTCTCAGAAAGAG CATTGT	ND
	CRISPR	contig05	213,971	214,094	1	AGAAGAAGTAACGGAAGA AGAAAAAGCGAAAGCCAA GGCG	ND
	CRISPR	contig05	230,560	230,693	2	GACAAATCTCAAAAAGAA GAGAA	+
	CRISPR	contig07	324,489	324,562	1	ATCATCATCATGGAGGACA CAAT	ND
	Cas cluster	contig12	8,853	10,442	2	cas3_TypeI, cas3_TypeI	-
	Cas cluster	contig14	24,319	27,849	2	cas3_TypeI, cas3_TypeI	-
***Bacillus* sp. GL1**	CRISPR	contig01	1,421,764	1,421,865	1	AGTTTAGGTTTCTTTTGAG AATGT	ND
	CRISPR	contig02	899,058	899,249	2	GTATGATTACCTTCCGCATG AGAA	ND
	CRISPR	contig03	631,716	631,823	1	AAACGTTTGTTTAAGATGT ATGTTCGG	ND
	CRISPR	contig04	58,448	58,516	1	TATAAGTTATATAACTTATAT TGA	ND
***Bacillus cereus* GL2**	CRISPR	contig01	559,196	559,289	1	TGAATTAATGCAACAAATT GCTCAG	ND
	CRISPR	contig01	1,281,819	1,281,927	1	TATATCAACGATTTTTTAAA TATATCG	ND
	CRISPR	contig01	2,888,091	2,888,198	1	TATATCTATCATAACTTACA ATATAT	ND
	CRISPR	contig01	3,295,305	3,295,438	2	GTTGATTTCTCTTCTTTTTG AGA	-
	CRISPR	contig01	3,343,896	3,344,000	1	AGCTTCAGCAGCTAGAAG GGTCGGT	ND
	Cas cluster	contig04	3,435	9,647	5	cas6_TypeI-III, cas8a1b_TypeIB, cas7b_TypeIB, cas5b_TypeIB, cas3_TypeI	-
	CRISPR	contig04	187,451	187,519	1	TATAAGTTATATAACTTATAT TGA	ND
***Bacillus wiedmannii* GL3**	CRISPR	contig01	3,890,544	3,890,774	3	AACAAGACGAAGCTCAGA AAAAAGCTG	ND
	CRISPR	contig01	4,320,165	4,320,344	2	TTCTTTTCTGTCTCAGTTTT TTCGTTCTTTTCTGTT	ND
	CRISPR	contig01	4,366,466	4,366,573	1	CCAATGTTAACGTAGTCTG GTCTTGCTGGTGCT	ND

*ND – not determined.
