# Supplementary figures and images for: Exploring the diversity and genomics of cultivable Bacillus-related endophytic bacteria from the medicinal plant Galium aparine L
Source: Front Microbiol. 2025 Jun 30;16:1612860. doi: 10.3389/fmicb.2025.1612860 (PMC12256460; doi:10.3389/fmicb.2025.1612860)

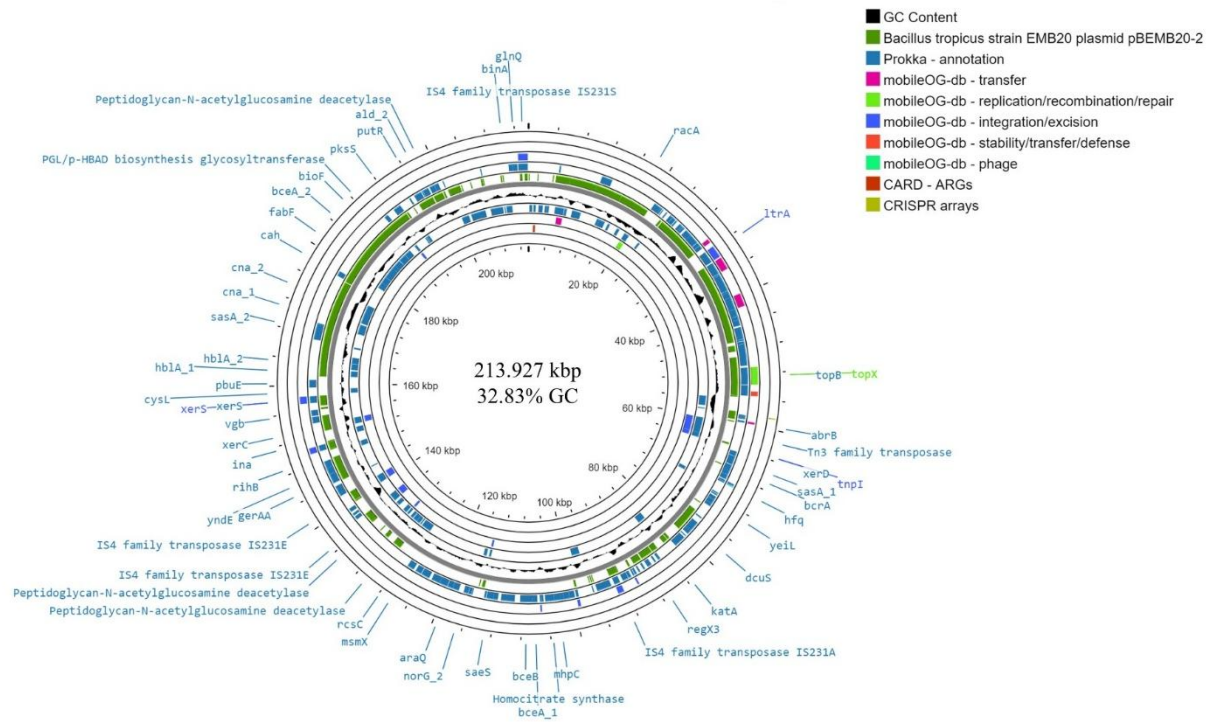

#### E. *Bacillus* sp. GL1, contig 4

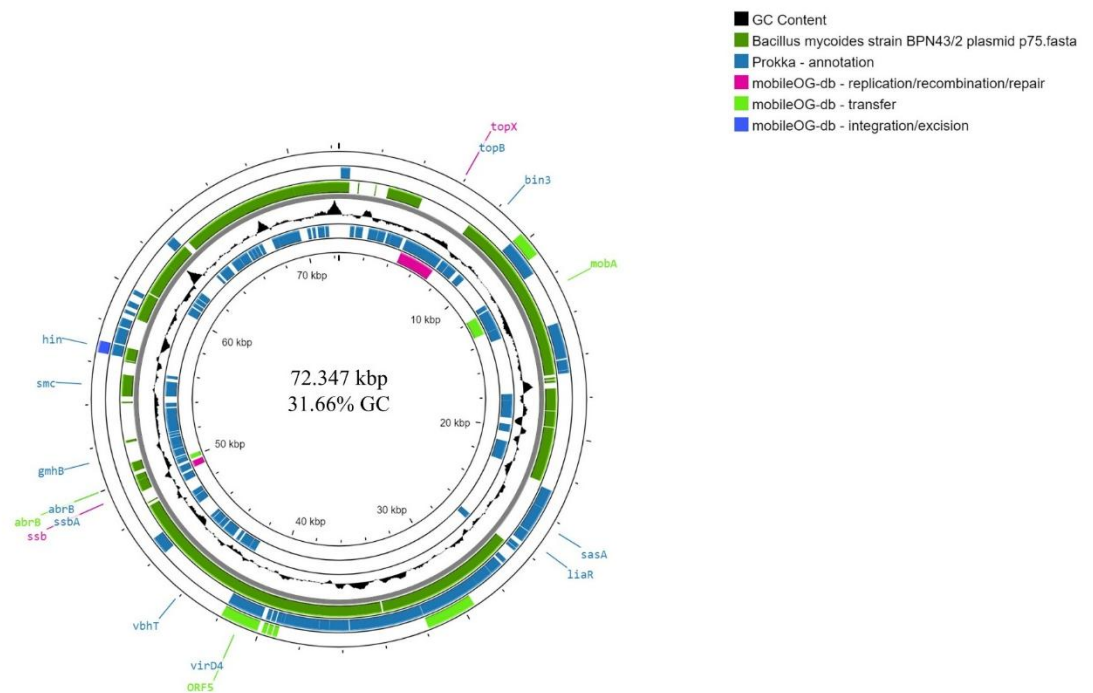

#### F. *Bacillus* sp. GL1, contig 6

Supplement: Supplementary file 2 [file Data_Sheet_2.pdf]
