# Supplementary material for: Exploring the diversity and genomics of cultivable Bacillus-related endophytic bacteria from the medicinal plant Galium aparine L
Source: Front Microbiol. 2025 Jun 30;16:1612860. doi: 10.3389/fmicb.2025.1612860 (PMC12256460; doi:10.3389/fmicb.2025.1612860)
Supplement: Supplementary file 3 [file Data_Sheet_3.pdf]

Figure S2. Distribution of different CAZymes subfamilies in *Galium aparine* L.'s endophytes

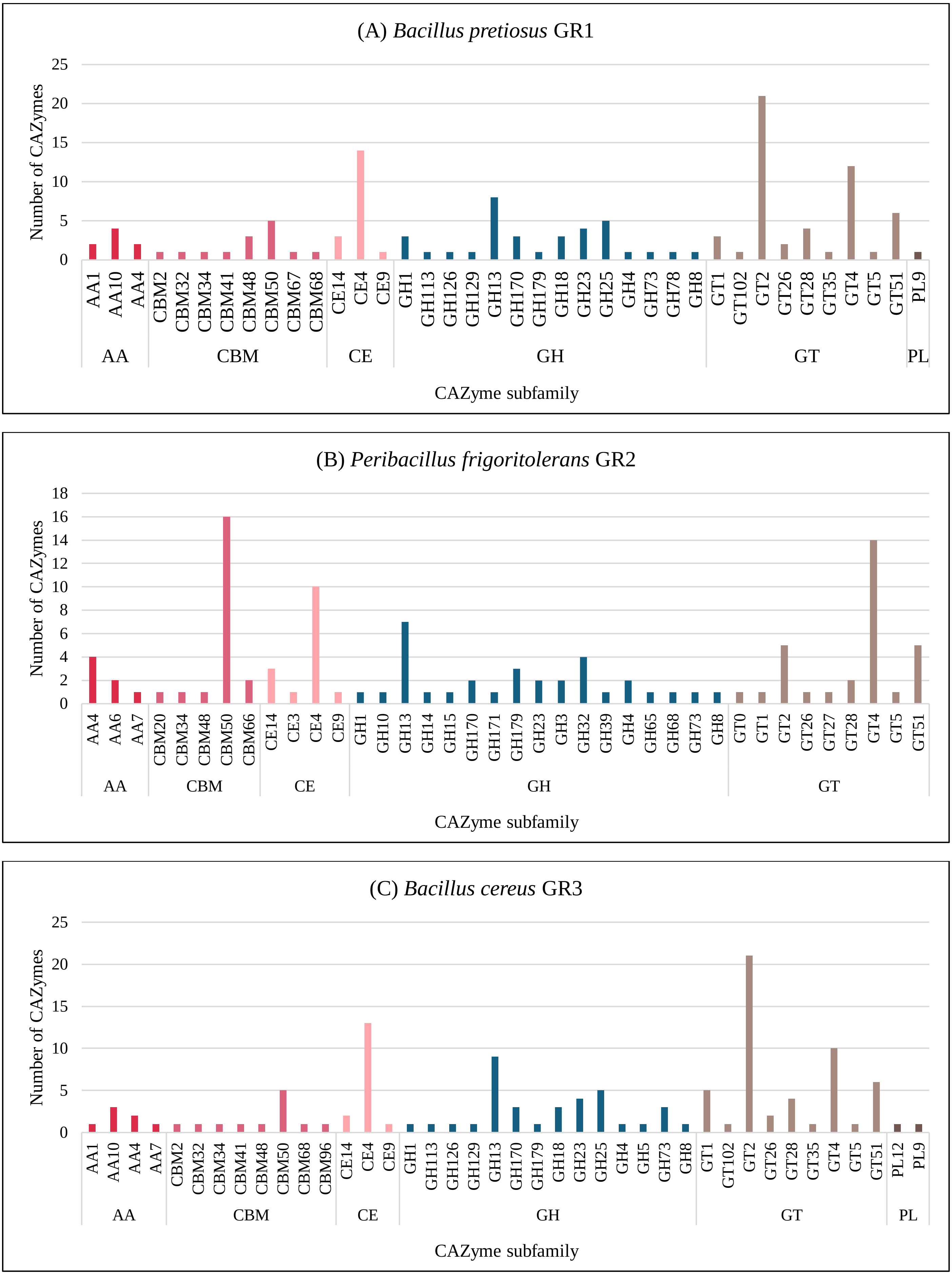

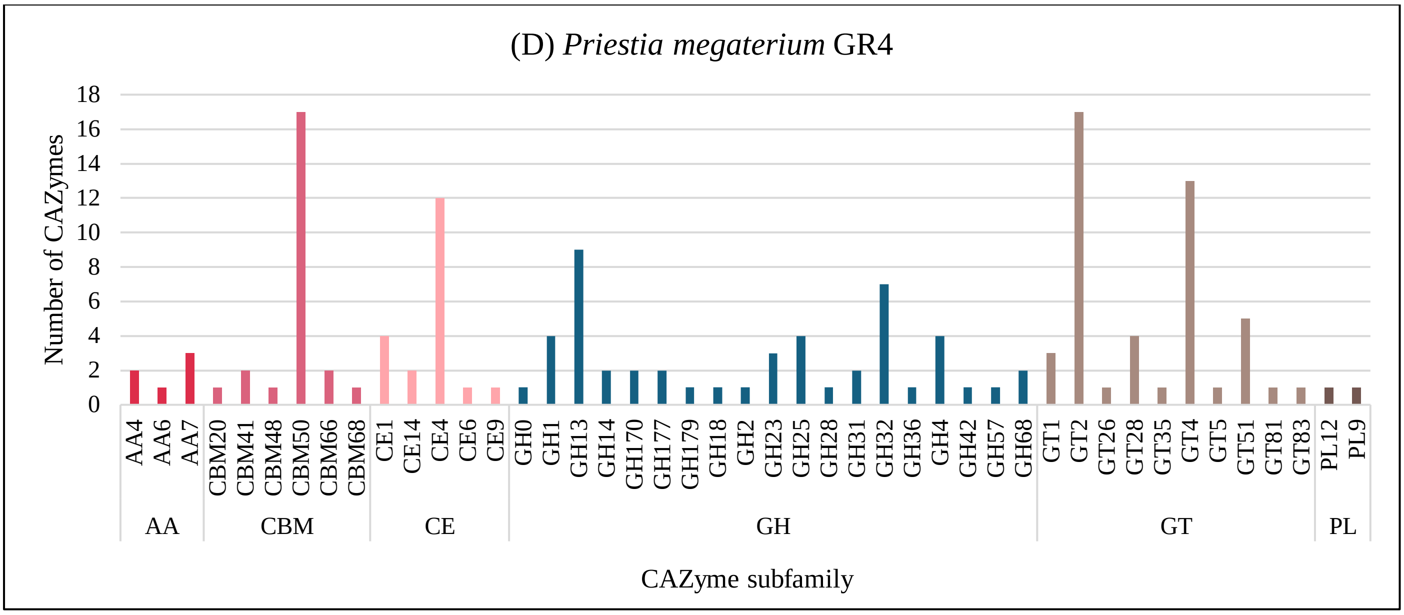
(D) *Priestia megaterium* GR4
Number of CAZymes
0 2 4 6 8 10 12 14 16 18
AA4 AA6 AA7 CBM20 CBM41 CBM48 CBM50 CBM66 CBM68 CE1 CE14 CE4 CE6 CE9 GH0 GH1 GH13 GH14 GH170 GH177 GH179 GH18 GH2 GH23 GH25 GH28 GH31 GH32 GH36 GH4 GH42 GH57 GH68 GT1 GT2 GT26 GT28 GT35 GT4 GT5 GT51 GT81 GT83 PL12 PL9
AA CBM CE GH GT PL
CAZyme subfamily

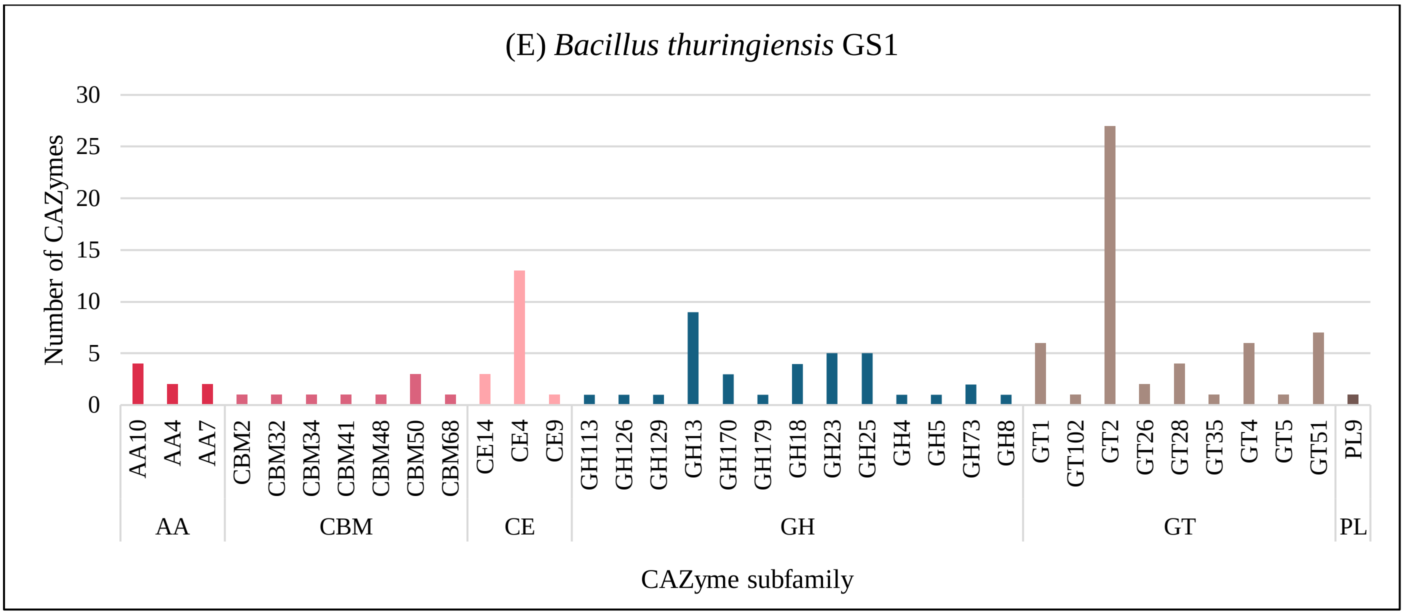
(E) *Bacillus thuringiensis* GS1
Number of CAZymes
0 5 10 15 20 25 30
AA10 AA4 AA7 CBM2 CBM32 CBM34 CBM41 CBM48 CBM50 CBM68 CE14 CE4 CE9 GH113 GH126 GH129 GH13 GH170 GH179 GH18 GH23 GH25 GH4 GH5 GH73 GH8 GT1 GT102 GT2 GT26 GT28 GT35 GT4 GT5 GT51 PL9
AA CBM CE GH GT PL
CAZyme subfamily

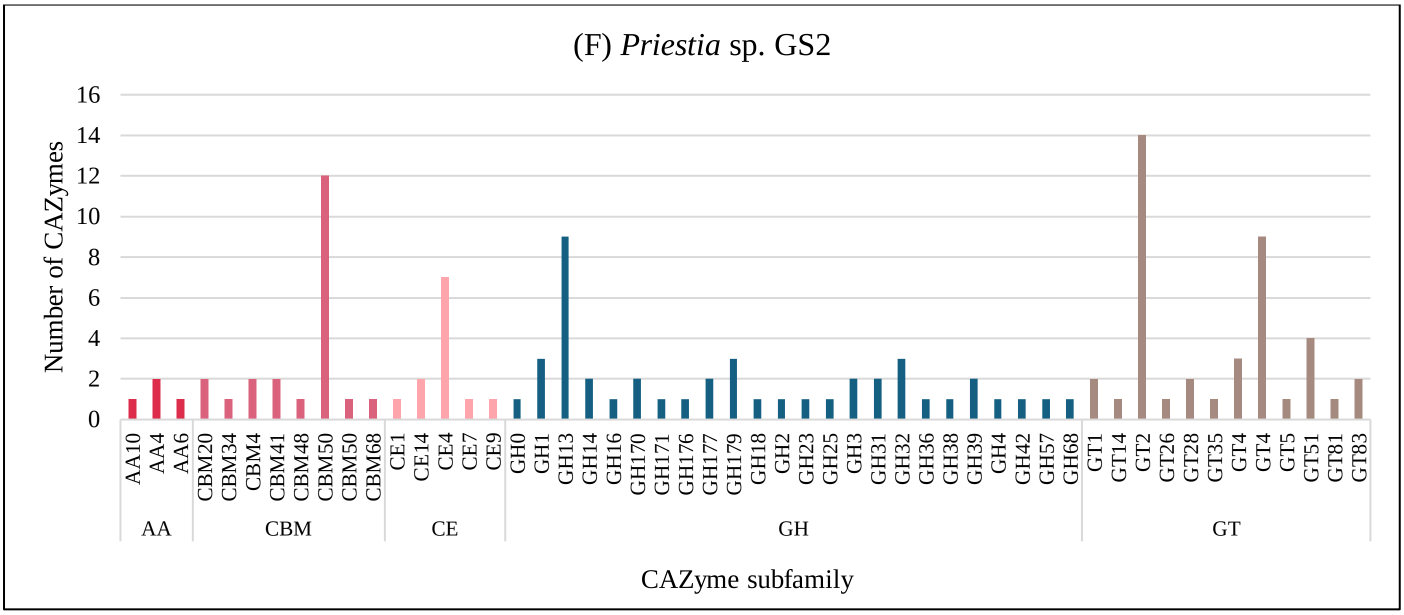
(F) *Priestia* sp. GS2
Number of CAZymes
0 2 4 6 8 10 12 14 16
AA10 AA4 AA6 CBM20 CBM34 CBM4 CBM41 CBM48 CBM50 CBM50 CBM68 CE1 CE14 CE4 CE7 CE9 GH0 GH1 GH13 GH14 GH16 GH170 GH171 GH176 GH177 GH179 GH18 GH2 GH23 GH25 GH3 GH31 GH32 GH36 GH38 GH39 GH4 GH42 GH57 GH68 GT1 GT14 GT2 GT26 GT28 GT35 GT4 GT4 GT5 GT51 GT81 GT83
AA CBM CE GH GT
CAZyme subfamily

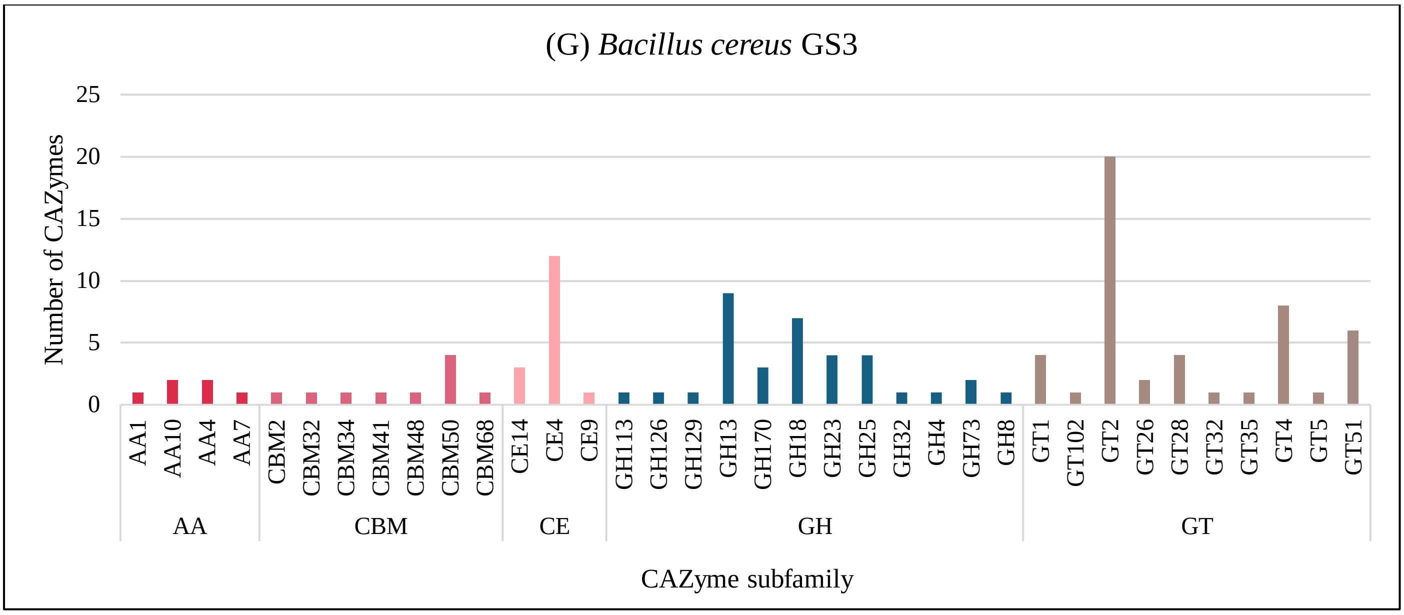
(G) Bacillus cereus GS3
Number of CAZymes
0
5
10
15
20
25
AA1
AA10
AA4
AA7
CBM2
CBM32
CBM34
CBM41
CBM48
CBM50
CBM68
CE14
CE4
CE9
GH113
GH126
GH129
GH13
GH170
GH18
GH23
GH25
GH32
GH4
GH73
GH8
GT1
GT102
GT2
GT26
GT28
GT32
GT35
GT4
GT5
GT51
AA
CBM
CE
GH
GT
CAZyme subfamily

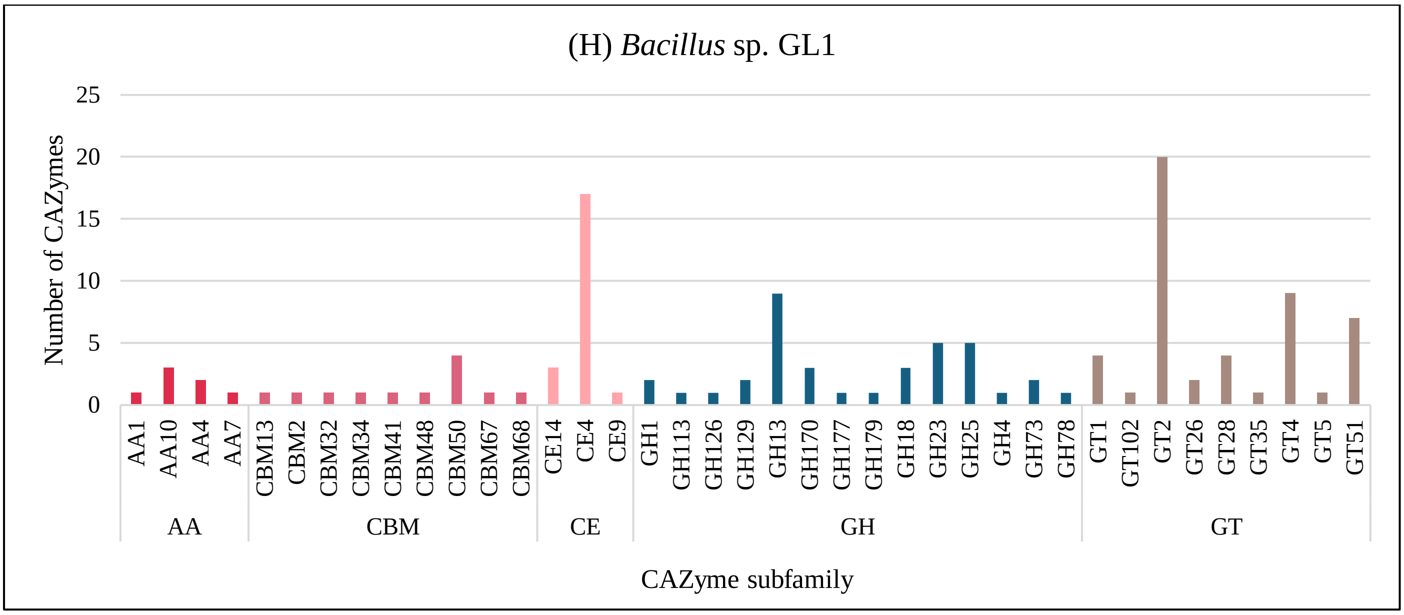
(H) Bacillus sp. GL1
Number of CAZymes
0
5
10
15
20
25
AA1
AA10
AA4
AA7
CBM13
CBM2
CBM32
CBM34
CBM41
CBM48
CBM50
CBM67
CBM68
CE14
CE4
CE9
GH1
GH113
GH126
GH129
GH13
GH170
GH177
GH179
GH18
GH23
GH25
GH4
GH73
GH78
GT1
GT102
GT2
GT26
GT28
GT35
GT4
GT5
GT51
AA
CBM
CE
GH
GT
CAZyme subfamily

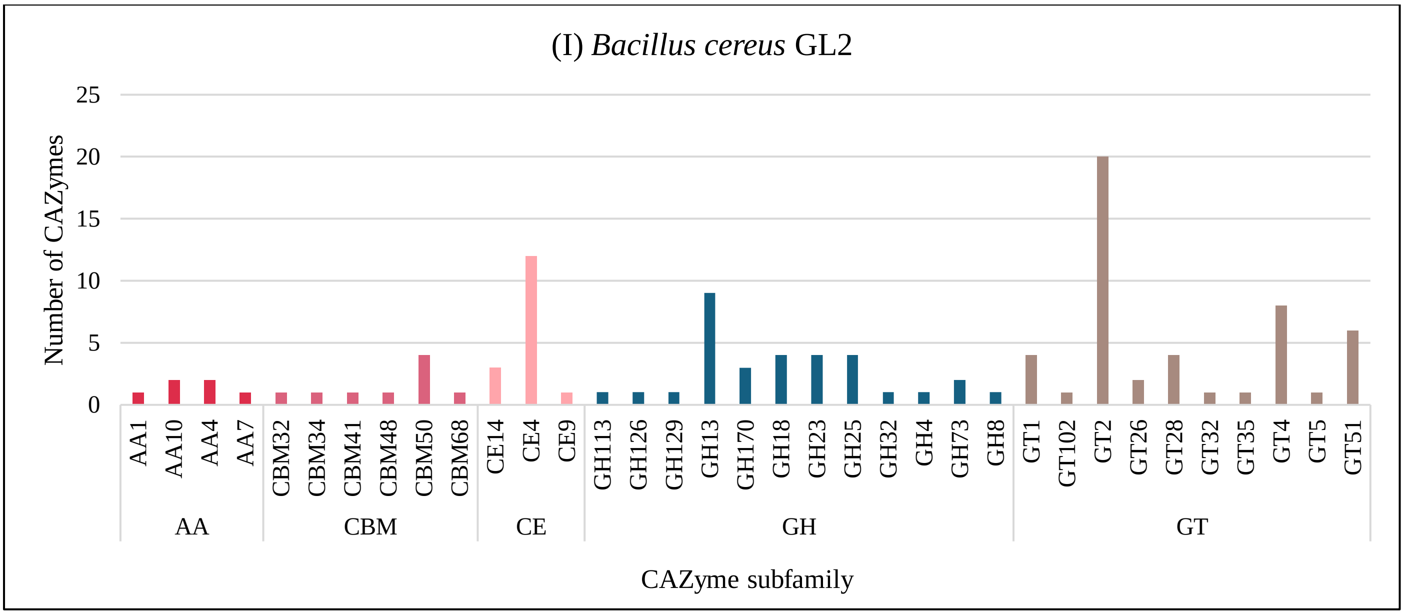
(I) Bacillus cereus GL2
Number of CAZymes
0
5
10
15
20
25
AA1
AA10
AA4
AA7
CBM32
CBM34
CBM41
CBM48
CBM50
CBM68
CE14
CE4
CE9
GH113
GH126
GH129
GH13
GH170
GH18
GH23
GH25
GH32
GH4
GH73
GH8
GT1
GT102
GT2
GT26
GT28
GT32
GT35
GT4
GT5
GT51
AA
CBM
CE
GH
GT
CAZyme subfamily

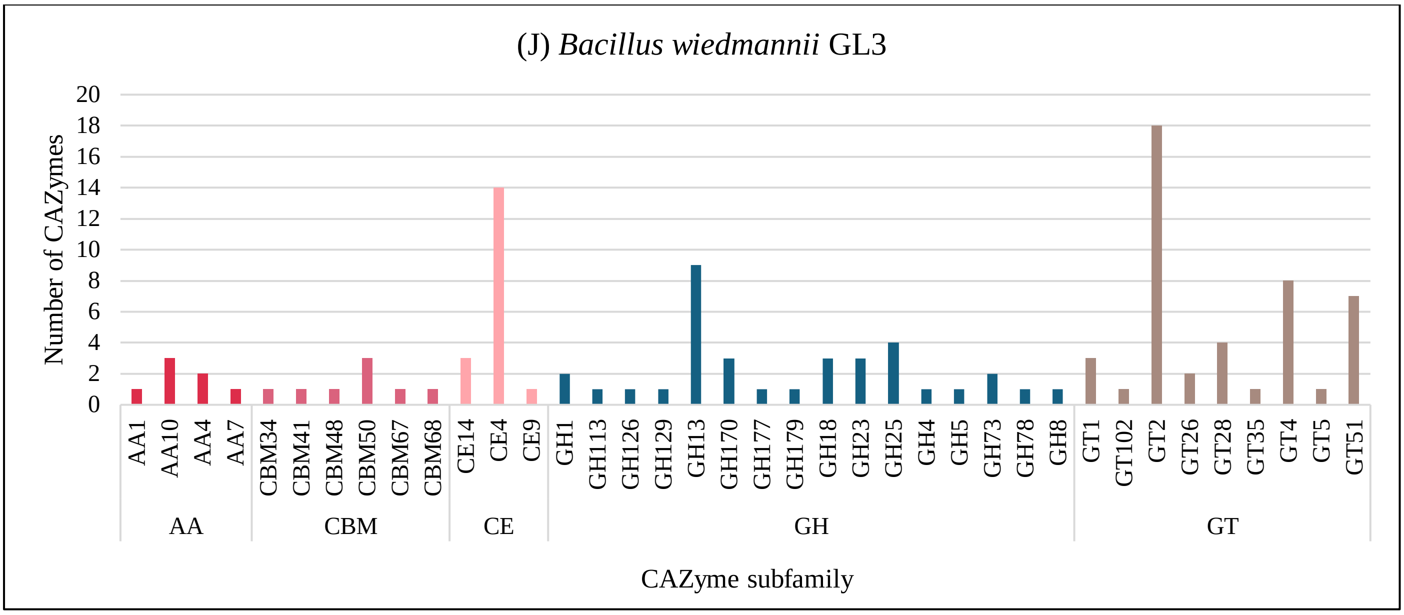
(J) Bacillus wiedmannii GL3
Number of CAZymes
20
18
16
14
12
10
8
6
4
2
0
AA1
AA10
AA4
AA7
CBM34
CBM41
CBM48
CBM50
CBM67
CBM68
CE14
CE4
CE9
GH1
GH113
GH126
GH129
GH13
GH170
GH177
GH179
GH18
GH23
GH25
GH4
GH5
GH73
GH78
GH8
GT1
GT102
GT2
GT26
GT28
GT35
GT4
GT5
GT51
AA
CBM
CE
GH
GT
CAZyme subfamily
