# Supplementary material for: Exploring the diversity and genomics of cultivable Bacillus-related endophytic bacteria from the medicinal plant Galium aparine L
Source: Front Microbiol. 2025 Jun 30;16:1612860. doi: 10.3389/fmicb.2025.1612860 (PMC12256460; doi:10.3389/fmicb.2025.1612860)
Supplement: Supplementary file 4 [file Data_Sheet_4.pdf]

Table S2. BGCs identified in genomes of *Galium aparine* L.'s bacterial endophytes

Strain	Region	Type	Start	End	Length (bp)	Most similar known cluster[1]	Similarity[2] (%)	Classified as
***Bacillus pretiosus* GR1**	1.1	LAP*	884,844	908,351	23,508	-	-	RiPP*
	1.2	NI-siderophore*	1,531,128	1,562,833	31,706	petrobactin	100	other
	1.3	NRP-metallophore, NRPS*	1,839,998	1,891,752	51,755	bacillibactin	85	NRPS
	1.4	NRPS	1,977,111	2,043,019	65,909	zwittermicin A	18	NRPS
	1.5	betalactone	2,049,638	2,074,875	25,238	fengycin	40	betalactone
	1.6	RiPP-like	2,132,316	2,142,651	10,336	-	-	RiPP
	1.7	RiPP-like	2,215,981	2,226,244	10,264	-	-	RiPP
	1.8	NRPS	2,241,558	2,288,568	47,011	-	-	NRPS
	1.9	terpene	3,027,322	3,049,175	21,854	molybdenum cofactor	17	terpene
	2.1	RRE-containing*, lassopeptide	30,236	52,967	22,732	-	-	RiPP
	2.2	NRPS, RRE-containing	80,053	126,217	46,165	-	-	NRPS
	6.1	lanthipeptide class II	1	13,212	13,212	-	-	RiPP
	6.2	RiPP-like, lanthipeptide class II	13,569	34,860	21,292	bacillicn CER074	25	RiPP
		Genome size (bp):	5,662,838	All (bp):	390,982		% of BGCs:	6.90
***Peribacillus frigoritolerans* GR2**	1	NRPS, betalactone	740,249	802,784	62,536	koranimine	87	hybrid
	2	betalactone	2,483,856	2,508,025	24,170	fengycin	46	betalactone
	3	T3PKS*	3,435,586	3,476,674	41,089	-	-	PKS*
	4	terpene	3,611,497	3,632,315	20,819	-	-	terpene
	5	lassopeptide	3,829,690	3,853,684	23,995	paeninodin	100	RiPP
	6	LAP*	4,600,549	4,624,084	23,536	-	-	RiPP
	7	terpene	5,246,419	5,268,464	22,046	-	-	terpene
	8	NI-siderophore	5,561,399	5,594,911	33,513	schizokinen	60	other
		Genome size (bp):	5,628,300	All (bp):	251,704		% of BGCs:	4.47
***Bacillus cereus* GR3**	1.1	lanthipeptide class I	374,807	403,179	28,373	-	-	RiPP
	1.2	terpene	1,906,148	1,928,001	21,854	molybdenum cofactor	17	terpene
	1.3	NRPS	2,715,519	2,762,535	47,017	-	-	NRPS
	1.4	RiPP-like	2,778,073	2,788,321	10,249	-	-	RiPP
	1.5	RiPP-like	2,910,112	2,920,432	10,321	-	-	RiPP
	1.6	betalactone	2,973,924	2,999,162	25,239	fengycin	40	betalactone
	1.7	NRPS	3,029,682	3,095,590	65,909	-	-	NRPS
	2.1	NRP-metallophore, NRPS	35,258	87,006	51,749	bacillibactin	85	NRPS
	2.2	NI-siderophore	365,026	396,733	31,708	petrobactin	100	other
	2.3	LAP	1,053,385	1,076,891	23,507	-	-	RiPP
	2.4	NRPS-like	1,842,854	1,886,435	43,582	-	-	NRPS
	3.1	sactipeptide	150,793	172,172	21,380	thuricin H	70	RiPP
	3.2	lanthipeptide class II, NRPS, T1PKS*	174,271	318,725	144,455	zwittermicin A	100	hybrid
		Genome size (bp):	5,972,447	All (bp):	525,343		% of BGCs:	8.80
***Priestia megaterium* GR4**	1.1	T3PKS	418,803	459,888	41,086	-	-	PKS
	1.2	phosphonate	1,163,027	1,180,448	17,422	-	-	other
	1.3	terpene	1,303,866	1,324,714	20,849	carotenoid	50	terpene
	2.1	NI-siderophore	814,874	849,449	34,576	synechobactin	23	other

	2.2	terpene	1,000,579	1,021,397	20,819	surfactin	13	terpene
	3.1	terpene	594,483	616,351	21,869	-	-	terpene
	8.1	RRE-containing, LAP	1	31,158	31,158	-	-	RiPP
		Genome size (bp):	5,689,305	All (bp):	187,779		% of BGCs:	3.30
**Bacillus thuringiensis GS1**	1.1	LAP	161,123	184,629	23,507	-	-	RiPP
	3.1	NRPS	466,897	513,907	47,011	-	-	NRPS
	3.2	RiPP-like	534,188	544,448	10,261	-	-	RiPP
	3.3	RiPP-like	609,627	619,956	10,330	-	-	RiPP
	3.4	betalactone	675,481	700,719	25,239	fengycin	40	betalactone
	4.1	NRPS	15,886	81,794	65,909	saccharothrixin	6	NRPS
	4.2	NRP-metallophore, NRPS	171,559	223,307	51,749	bacillibactin	85	NRPS
	4.3	NI-siderophore	508,077	539,784	31,708	petrobactin	100	other
	5.1	lanthipeptide class II	374,480	397,638	23,159	cerecidin	94	RiPP
	6.1	triceptide, sactipeptide, NRPS, T1PKS	5,007	135,299	130,293	zwittermicin A	100	hybrid
	6.2	RiPP-like	183,339	195,558	12,220	-	-	RiPP
	6.3	lanthipeptide class II	436,009	459,278	23,270	gramicidin S	15	RiPP
	7.1	NRPS-like	91,874	135,455	43,582	-	-	NRPS
	10.1	terpene	49,327	71,180	21,854	molybdenum cofactor	17	terpene
		Genome size (bp):	5,957,201	All (bp):	520,092		% of BGCs:	8.73
***Priestia* sp. GS2**	1.1	NI-siderophore	895,315	929,315	34,001	schizokinen	40	other
	1.2	RRE-containing, terpene	1,973,448	2,007,931	34,484	-	-	terpene
	1.3	opine-like metallophore	2,184,621	2,206,730	22,110	bacillopaline	100	other
	1.4	T3PKS	2,350,062	2,391,150	41,089	-	-	PKS
	1.5	lassopeptide	2,692,520	2,716,439	23,920	paeninodin	60	RiPP
	1.6	terpene	3,100,637	3,121,473	20,837	carotenoid	50	terpene
	2.1	cyclic lactone autoinducer, RiPP-like	47,718	68,465	20,748	-	-	RiPP
		Genome size (bp):	4,167,990	All (bp):	197,189		% of BGCs:	4.73
**Bacillus cereus GS3**	1.1	LAP	37,940	61,446	23,507	-	-	RiPP
	1.2	NRPS, transAT-PKS*	590,849	654,100	63,252	tauramamide	9	hybrid
	1.3	NI-siderophore	723,995	755,703	31,709	petrobactin	100	other
	1.4	NRP-metallophore, NRPS	1,067,542	1,119,290	51,749	bacillibactin	85	NRPS
	1.5	NRPS	1,202,310	1,268,218	65,909	-	-	NRPS
	1.6	betalactone	1,280,041	1,305,279	25,239	fengycin	40	betalactone
	1.7	RiPP-like	1,359,697	1,370,017	10,321	-	-	RiPP
	1.8	RiPP-like	1,414,973	1,425,239	10,267	-	-	RiPP
	1.9	NRPS	1,438,181	1,485,197	47,017	-	-	NRPS
	1.10	terpene	2,251,563	2,273,416	21,854	molybdenum cofactor	17	terpene
	4.1	NRPS-like	42,808	86,389	43,582	-	-	NRPS
	6.1	RiPP-like	126,436	139,371	12,936	-	-	RiPP
	7.1	NRPS, NI-siderophore	19,155	82,675	63,521	schizokinen	60	hybrid
		Genome size (bp):	5,705,209	All (bp):	470,863		% of BGCs:	8.25
***Bacillus* sp. GL1**	1.1	NRPS, arylpolyene	129,922	191,466	61,545	-	-	NRPS
	1.2	NRPS	204,328	251,926	47,599	-	-	NRPS
	1.3	betalactone	482,594	507,831	25,238	fengycin	40	betalactone
	1.4	RiPP-like	568,780	579,100	10,321	-	-	RiPP

	1.5	RiPP-like	630,136	640,408	10,273	-	-	RiPP
	1.6	terpene	1,469,814	1,488,834	19,021	molybdenum cofactor	17	terpene
	1.7	lassopeptide	1,607,505	1,631,415	23,911	paeninodin	100	RiPP
	2.1	LAP	934,265	957,772	23,508	-	-	RiPP
	2.2	NI-siderophore	1,581,300	1,613,006	31,707	petrobactin	100	other
	2.3	NRP-metallophore, NRPS	1,971,108	2,022,852	51,745	bacillibactin	85	NRPS
		Genome size (bp):	6,101,238	All (bp):	304,868		% of BGCs:	5.00
	1.1	NRPS, transAT-PKS, NRPS-like	1	31,295	31,295	tauramamide	9	hybrid
	1.2	NI-siderophore	101,190	132,898	31,709	petrobactin	100	other
	1.3	NRP-metallophore, NRPS	428,404	480,152	51,749	bacillibactin	85	NRPS
	1.4	NRPS	563,172	629,080	65,909	-	-	NRPS
	1.5	betalactone	640,903	666,141	25,239	fengycin	40	betalactone
	1.6	RiPP-like	720,559	730,879	10,321	-	-	RiPP
	1.7	RiPP-like	775,835	786,101	10,267	-	-	RiPP
Bacillus cereus	1.8	NRPS	799,043	846,059	47,017	-	-	NRPS
**GL2**	1.9	terpene	1,612,388	1,634,241	21,854	molybdenum cofactor	17	terpene
	2.1	LAP	304,892	328,398	23,507	-	-	RiPP
	2.2	NRPS	859,397	887,577	28,181	-	-	NRPS
	3.1	NRPS-like	167,323	210,904	43,582	-	-	NRPS
	4.1	RiPP-like	123,354	136,289	12,936	-	-	RiPP
	5.1	NRPS, NI-siderophore	72,307	135,827	63,521	schizokinen	60	hybrid
	8.1	NRPS	1	2,464	2,464	-	-	NRPS
		Genome size (bp):	5,704,787	All (bp):	469,551		% of BGCs:	8.23
	1.1	terpene	1,951,622	1,973,475	21,854	molybdenum cofactor	17	terpene
	1.2	NRPS	2,731,084	2,778,088	47,005	-	-	NRPS
	1.3	RiPP-like	2,795,001	2,805,273	10,273	-	-	RiPP
	1.4	RiPP-like	2,849,807	2,860,124	10,318	-	-	RiPP
	1.5	betalactone	2,923,391	2,948,629	25,239	fengycin	40	betalactone
	1.6	NRP-metallophore, NRPS	3,084,284	3,136,045	51,762	bacillibactin	85	NRPS
Bacillus	1.7	NI-siderophore	3,494,013	3,525,720	31,708	petrobactin	100	other
***wiedmannii* GL3**	1.8	LAP	4,138,422	4,161,929	23,508	-	-	RiPP
	1.9	LAP	5,013,050	5,035,984	22,935	-	-	RiPP
	2.1	NRPS	180,419	236,640	56,222	-	-	NRPS
	2.2	RiPP-like	338,072	350,943	12,872	-	-	RiPP
	2.3	NRPS	388,778	436,376	47,599	-	-	NRPS
	3.1	CDPS*	1	16,059	16059	pulcherriminic acid	66	other
		Genome size (bp):	5,989,811	All (bp):	377,354		% of BGCs:	6.30

* CDPS - tRNA-dependent cyclodipeptide synthases; LAP - linear azol(in)e-containing peptides; NI-siderophore - NRPS-independent, IucA/IucC-like siderophores; NRP-metallophore - non-ribosomal peptide metallophores; NRPS - non-ribosomal peptide synthetase; PKS - polyketide synthase: RiPP - ribosomally synthesised and post-translationally modified peptide product; RRE-containing - RRE-element containing cluster; T1PKS - type I PKS; T3PKS - type III PKS; transAT-PKS – PKS with trans-AT domain.

[1]Most similar known compound with a cluster in MIBiG (Minimum Information about a Biosynthetic Gene cluster) database

[2] % of genes within the closest known compound that have a significant BLAST hit to genes within the current region
